# Molecular modeling and quantum chemical calculation study of derived peptides from a Bowman-Birk inhibitor in complexes with trypsin

**DOI:** 10.1007/s00894-026-06931-0

**Published:** 2026-09-05

**Authors:** Luiz F. M. A. Benício, Érica C. M. Nascimento, Sonia M. de Freitas, João B. L. Martins

**Affiliations:** 1 Department of Pharmacy, Faculty of Health Sciences, University of Brasília, Brasília, DF 70910-900 Brazil; 2 Computational Chemistry Laboratory, Institute of Chemistry, University of Brasilia, Brasilia, DF 70910-900 Brazil; 3 Biophysics Laboratory, Institute of Biological Science, University of Brasilia, Brasilia, DF 70910-900 Brazil

**Keywords:** Bowman-Birk inhibitor, Molecular modeling, Quantum chemical calculation

## Abstract

**Context:**

Bowman-Birk inhibitors (BBIs) are cysteine-rich proteins that inhibit trypsin and chymotrypsin proteases. Derived from soybeans, these natural BBIs have been identified as potential anticarcinogenic agents. Recent studies of the black-eyed pea trypsin/chymotrypsin inhibitor (BTCI) demonstrated that peptides derived from the BBI family may retain their structure and inhibitory activity. Therefore, this study aimed to investigate the effects of solvent on BTCI-derived peptides and their inhibitory activity against trypsin. The results show that cyclic peptides exhibit reduced conformational flexibility, enhanced reactivity (narrower HOMO–LUMO gap), and LUMO localized at the disulfide bonds, in contrast to the linear peptide Ptry6. Non-covalent interactions were evaluated using the Independent Gradient Model based on Hirshfeld partitioning (IGMH). Molecular electrostatic potential maps and the IGMH confirmed that the cyclic architecture concentrates electropositive donor regions at key residues (Thr2, Lys3, Ser4, and Ile5), whereas Ptry6 displays only weak van der Waals contacts. Docking studies against trypsin showed strong hydrogen-bond and electrostatic interactions between cyclic peptides and residues involved in the catalytic mechanism. Predicted inhibition constants obtained from docking studies closely match experimental data. These findings suggest that electronic redistribution upon solvation governs peptide–trypsin affinity, highlighting Ptry9L as the most promising BTCI-derived inhibitor for further biotechnological development.

**Methods:**

BTCI peptides were studied using quantum-chemical calculations and molecular docking. Three peptides—a cyclic nonapeptide in both L- and D-configurations (Ptry9L, Ptry9D) and a linear hexapeptide (Ptry6)—were optimized in vacuo and in implicit aqueous solvent using density functional theory at B3LYP-D3/Def2-SVP and B3LYP-D3/6-311+G(d,p) levels with D3 dispersion correction. Frontier molecular orbital analyses (HOMO–LUMO gaps and orbital localization) and IGMH revealed that the disulfide bond stabilizes cyclic peptides.

**Supplementary information:**

The online version contains supplementary material available at https://doi.org/10.1007/s00894-026-06931-0.

## Introduction

Trypsin inhibitors are found in many living organisms and are recognized as serine protease inhibitors [1–3]. Bowman-Birk inhibitors (BBIs) are cysteine-rich proteins widely distributed in monocotyledonous and dicotyledonous species [4, 5]. The BBIs are able to inhibit trypsin and chymotrypsin proteases independently and simultaneously [6–8]. Natural soybean-derived BBIs with inhibitory activity against trypsin and chymotrypsin are potential anticarcinogenic agents in vivo and in vitro systems [9–11] due to trypsin and chymotrypsin-like proteases involved in cancer processes [10, 12–14]. BBI acts as an analog of the natural substrate, competing for the active site of trypsin or trypsin-like protein, generating an inactivated ligand-enzyme complex [15].

The black-eyed pea trypsin/chymotrypsin inhibitor (BTCI) is a globular BBI protein, consisting of an 83-residue polypeptide chain, and it is isolated from the *Vigna unguiculata* (*cv Seridó*) seeds [16]. BTCI was well-characterized as a potential molecule with pharmacological applications, e.g., as an anticarcinogenic agent in breast cancer cells by inhibition of trypsin-*like*, chymotrypsin-*like*, and caspase-*like* specific protease sites of the *20S* proteasome; as an enhancer of guanylin-induced natriuresis in isolated rat kidney assays, protecting its degradation via chymotrypsin-*like* proteases; and as a potential antihypertensive agent by hemodynamic and cardiovascular effects in Wistar and SHR rats via intravenous and gavage administrations [9, 12, 14, 17–19].

The structure of BTCI, solved by X-ray crystallography in a binary complex with *β*-trypsin and a ternary complex with chymotrypsin and trypsin, folds into two similar subdomains [4, 20]. Each subdomain is formed by antiparallel *β*-sheets that are stabilized by seven conserved disulfide bonds [4]. The subdomain containing the Lys residue inhibits trypsin, while the subdomain with the Phe residue inhibits chymotrypsin [4, 11]. The interaction between BTCI and trypsin is an endothermic, spontaneous process with positive entropic change [21, 22]. This process is accompanied by an entropy change resulting from the displacement of water molecules at the complex interface [21, 22]. BTCI interactions with trypsin are mediated by hydrogen bonds and a salt bridge between Lys26 and Asp189, respectively [4]. Residues Asp189, Ser190, Gly193, Ser195, and Ser210 are key to enzyme inhibition; some form the catalytic pocket, others the oxyanion hole, and others the catalytic triad [4, 23].

The S1 serine proteases, trypsin family of proteins, have many inhibitors, including natural and synthetic ones [24]. Due to their intensive and precise functions, these classes of enzymes have evolved in the setup of several illnesses, such as Alzheimer’s disease, neoplastic diseases, and blood system regulatory dysfunction [25]. Applications of BTCI and others demonstrate that peptides derived from the BBI family possibly retain their inhibitory activity and structure [23, 26–28]. The rational design of BTCI-derived peptides and the study of their structure and function are promising strategies for obtaining natural molecules for biotechnological purposes [19]. A peptide formed by nine amino acid residues named Ptry9, consisting of a Cys-Thr-Lys-Ser-Ile-Pro-Pro-Gln-Cys sequence, was selected from the structure of BTCI in complex with trypsin [29]. This amino acid sequence corresponds to a cyclic structure, maintained by a disulfide bond, and has two conformations, d-amino acid (Cys1D) and l-amino acid (Cys1L), named Ptry9D and Ptry9L, respectively [29]. Ptry9L exhibits complete inhibition of trypsin at a concentration of 50 µM [29]. Another related peptide derived from BTCI is Ptry6, a non-cyclic peptide with six residues (PDB code 6EAX). Due to its structural characteristics, the activity of Ptry6 is lower than that of both conformers of Ptry9 [29]. The amino acid sequence of Ptry6 (Cys-Thr-Lys-Ser-Ile-Cys) is similar to that of Ptry9, but it lacks two proline and one glutamine residues at positions six to eight in the Ptry9 sequence. The reduction in sequence length of Ptry6 and the absence of a disulfide bond result in a non-cyclic peptide, which exhibits no inhibitory activity against trypsin.

In this study, density functional theory was employed to obtain the electronic structure, molecular orbitals, and the Independent Gradient Model based on the Hirshfeld partition (IGMH) of the molecular density for BTCI-derived peptides. Subsequently, we performed molecular docking simulations between the optimized peptide structures and the trypsin enzyme (PDB codes 6E5M and 6EAT). The use of optimized peptide structures, obtained under quantum-mechanical methods, allows for the correlation of physicochemical and electronic properties determined at the electronic-structure level. Overall, this work provides insights into the energetic contributions underlying the inhibitory activity of BTCI-derived peptides against trypsin.

## Computational details

### Electronic structure

First, the structures of the BTCI-derived peptides, Ptry9D, Ptry9L, and Ptry6, were obtained from the *Protein Data Bank* (PDB) deposited under the codes 6E5M, 6EAT, and 6EAX [29], and checking and protonation of the residues were performed using GaussView v. 4.1.2 [30].

The peptides were previously analyzed using high-level quantum-chemistry methods to better understand the intramolecular interactions and molecular properties involved in complex formation with trypsin [31]. BTCI-derived peptide geometric structures were optimized in vacuum, considering the solvent effects using the implicit polarizable continuum model (PCM) in water [32]. In this model, the electrostatic contribution of the solvent over the accessible surface of the molecule of interest is considered, rather than explicitly modeling individual solvent molecules [32, 33]. In density functional theory (DFT), the Becke–three-parameter–Lee–Yang–Parr (B3LYP) hybrid functional [34–36] and the Def2-SVP and 6–311+G(d,p) basis sets were considered to calculate the electronic properties and determine the system’s minimum energy [37]. The Grimme D3 empirical dispersion term was included to properly account for dispersion interactions [38, 39]. Results without Grimme D3 empirical dispersion term are available in the Supplementary Information file (Tables S1-S3 and Figures S1-S4). Electronic structure calculations were performed using the Gaussian16 package, version C.01 [40], and the results were visualized with Chemcraft software, version 1.8, build 682 [41]. Molecular properties such as frontier molecular orbital (FMO) localization, *Δ*_FMO_ (HOMO–LUMO), and the Independent Gradient Model based on Hirshfeld (IGMH) partition [42] were used as indicators of the inhibitory tendency of these peptides and to account for non-covalent interactions. The Cartesian coordinates of the optimized geometries are available in the Supplementary Information file (Tables S4-S9).

### Docking study

A molecular docking study was performed using crystallographic structures of the cyclic nonapeptide derived from the BTCI inhibitor, bound to beta-trypsin, from PDB entries 6E5M, 6EAT, and 6EAX. The 6EAX structure was excluded from the analysis because the trypsin-bound peptide, Ptry6, lacked inhibitory activity in vitro. The trypsin structures in 6E5M and 6EAT were retained for docking studies, since Ptry9 inhibits trypsin at 50 µM [29]. Both 6E5M and 6EAT exhibit high-quality X-ray diffraction data (resolution < 2.00 Å) and complete residue/atom models, making them ideal for accurate molecular docking. The 6E5M and 6EAT structures were prepared by removing all crystallographic water molecules and buffer components. Residue protonation states were then assigned at pH 7.0 using the PROPKA 3.0 protocol [43]. In both models, the catalytic triad remained structurally intact and correctly protonated, ensuring a reliable starting point for subsequent docking simulations.

Molecular docking was performed using AutoDock 4.2.6 [44] with the AutodockTools protocol. Docking parameters were optimized using the Lamarckian Genetic Annealing (LGA) algorithm with genetic algorithm solutions set to 100, energy assessments set to 40,000,000, and a population size of 150. Grid parameters used in this study were set for each crystallographic structure. The 6EAT grid box was centered at 14.50, 57.48, and 146.49 with grid sizes of 16.50 Å, 21.00 Å, and 19.50 Å, while the 6E5M grid box was centered at 62.87, 166.80, and 5.91 with grid sizes of 16.50 Å, 19.50 Å, and 21.75 Å. The region analyzed comprised the trypsin active site corresponding to the His57, Asp102, and Ser195 residues, and other residues in the vicinity, such as Ser190, Asp189, and Gly193 [4, 11, 26]. Docking interactions were analyzed with VMD and Discovery Studio Visualizer [45, 46].

## Results and discussion

### Electronic structure of peptides

**Fig. 1:**
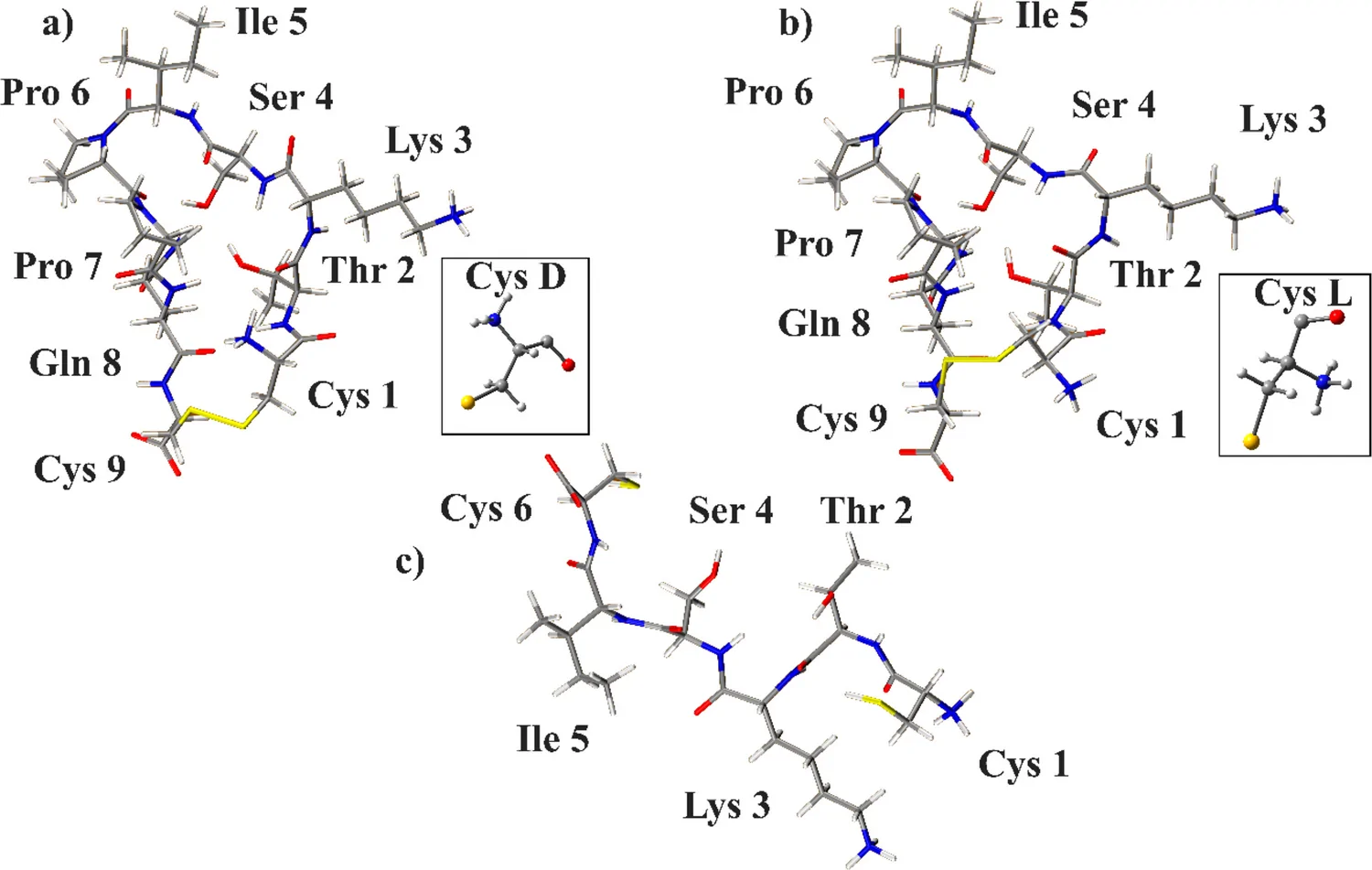
Optimized structures of peptides derived from BTCI using B3LYP-D3/6–311+G(d,p). **a**) Ptry9D, with Cys1 structure represented by Cys D highlighted; **b**) Ptry9L, with zoom in Cys1 structure represented by Cys L highlighted; and **c**) Ptry6 structure

Root mean square deviation (RMSD) analysis was performed to evaluate conformational differences between the optimized structures and the original PDB structures. This data consists of the structural displacements of the models compared to the PDB structures, which is essential, since the optimized structures from DFT serve as a starting point for molecular docking calculations. The results are summarized in Table 1. Both Ptry9L and Ptry9D peptides exhibit a cyclic folding, as shown in Fig. 1. This cyclic conformation of these solvated peptides restricts their degrees of freedom, limiting structural deviations. Consequently, RMSD values remained below 1.08 Å, indicating structural similarity. For further insights, we analyzed the results without the D3 dispersion correction (Tables S1 and S2 in the Supplementary information). The results demonstrate that this correction term impacts the final geometry, specifically the RMSD without D3, which increases considerably to 1.33 Å.

**Table 1:** RMSD values (Å) between the crystallographic structure and geometries of the obtained peptides using the B3LYP-D3/Def2-SVP and B3LYP-D3/6–311+G(d,p) in vacuum and implicit solvent PCM methods

	Conformation	Rotatable bonds	Def2-SVP		6–311+G(d,p)	
			Vacuum	Solvent	Vacuum	Solvent
Ptry9D	Cyclic	17	1.08	0.60	0.65	0.53
Ptry9L	Cyclic	17	0.87	0.50	0.97	0.68
Ptry6	Non-cyclic	32	2.98	2.64	2.56	1.70

As expected, the implicit solvent environment exerts a significant influence on the conformational changes of the peptides, as directly reflected in the RMSD values. Among the analyzed structures, Ptry6 showed the highest RMSD in both methods due to its open-chain configuration and the greater number of rotationally free bonds. In general, Def2-SVP and 6–311+G(d,p) optimizations yielded lower RMSD values of less than 1.08 Å. Additionally, the presence of solvent resulted in lower RMSD values across all structures, indicating greater conformational stability. However, 6–311+G(d,p) has the smallest RMSD values. Therefore, the B3LYP-D3/6–311+G(d,p)-optimized structures in the solvent were selected for subsequent property analyses and molecular docking studies.

Another important structural aspect of these peptides is their size, defined as the longest axis, i.e., the distance between the two most distant atoms on the same plane. Molecular dimensions directly dictate the accessibility to the trypsin active site and, therefore, are an important parameter for estimating inhibitory potency.

The parameters indicative of small-molecule reactivity, such as the dimensions of each molecule and the HOMO–LUMO energy gap (*Δ*_FMO_), are shown in Table 2 [47]. Given the crucial role of solvents in biological systems, variations in inhibitor size are expected under solvation conditions. Therefore, an implicit solvation model was used. Among the two electron density functionals, both the HOMO–LUMO energy gap (*Δ*_FMO_) and molecular dimensions exhibited trends consistent with the RMSD analysis: Ptry9D and Ptry9L displayed similar values, whereas Ptry6 showed a significant deviation. Notably, Ptry6 showed the largest one-dimensional molecular size and the widest HOMO–LUMO gap, consistent with a more flexible, open-chain structure.

**Table 2:** Calculated molecular size (Å) and gap energies (kcal/mol) at B3LYP-D3/Def2-SVP and B3LYP-D3/6–311+G(d,p) level using PCM implicit solvent model for Ptry9D, Ptry9L, and Ptry6 molecules

	*Def2-SVP solvent*		*6–311*+*G(d,p) solvent*	
	*Size*	*Gap (HOMO–LUMO)*	*Size*	*Gap (HOMO–LUMO)*
*Ptry9D*	17.96	117.44	17.81	119.95
*Ptry9L*	18.01	108.43	18.31	114.19
*Ptry6*	17.80	115.49	19.03	121.98

The B3LYP-D3/Def2-SVP-optimized structures exhibited energy gaps (*Δ*_FMO_) below 90.00 kcal/mol. In contrast, the B3LYP-D3/6–311+G(d,p)-optimized structures showed energy gaps approximately 50 kcal/mol greater than those obtained with Def2-SVP. Among both electron density functionals, the cyclic peptides Ptry9D and Ptry9L showed lower *Δ*_FMO_ values compared to the acyclic peptide Ptry6. In addition, Ptry9L showed the smallest gap, suggesting greater electronic stability for the cyclic conformations.

**Fig. 2:**
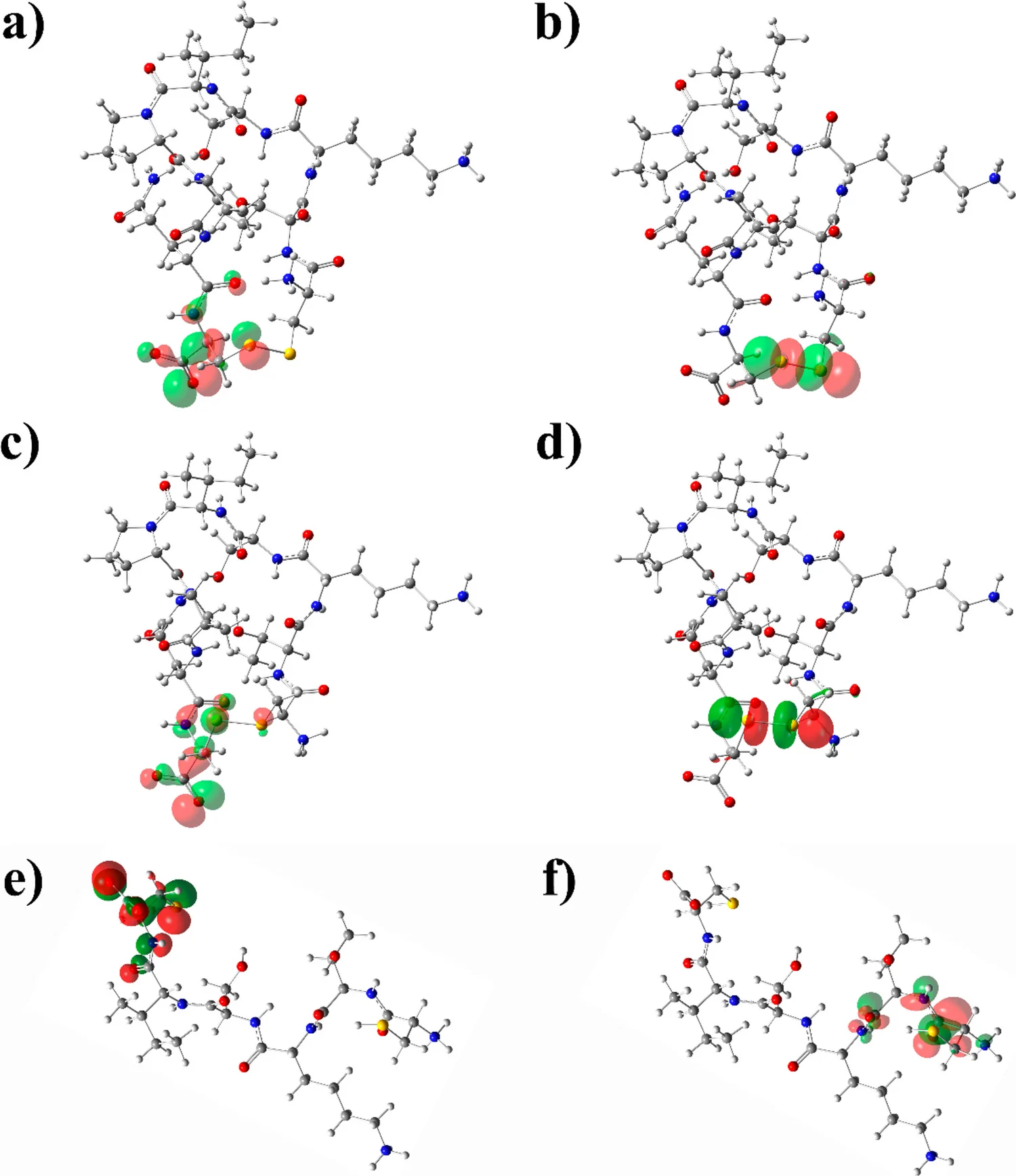
Molecular orbitals HOMO (left) and LUMO (right) of **a**, **b** Ptry9D, **c**, **d** Ptry9L, and **e**, **f** Ptry6 for the B3LYP-D3/6–311+G(d,p) in the solvent PCM model. Atom color code: navy (N), gray (C), red (O), yellow (S), and white (H). Red and green colors are used to represent orbital phases

Therefore, a higher reactivity tendency is expected from the cyclic peptides Ptry9D and Ptry9L. In this context, the experimental data showed that cyclic BTCI-derived peptides are important for trypsin inhibition [48], which may be related to the difference between Ptry9L/Ptry9D and Ptry6. Figure 2 shows the distribution of the frontier molecular orbitals (FMOs), providing insight into differences in electronic properties under the solvation model. The overall orbital distribution remains conserved in both electron density functionals, indicating that these parameters do not significantly alter the spatial localization of the FMOs.

The HOMO orbitals are predominantly distributed in the C-terminus of the peptides (COO^−^) over the Cys9 residue in Ptry9D and Ptry9L, and over Cys6 in Ptry6, with potential electron-donor character (Fig. 2a). In the crystallographic complex 6EAT, the Ptry9L molecule exhibits two hydrogen bonds with adjacent water molecules, with the HOMO orbital distribution localized in this region, thereby contributing to the donor potential and stabilization of the crystallographic structure [29]. In contrast, the LUMO orbital is predominantly located in the Cys1–Cys9 disulfide bond of both Ptry9D and Ptry9L. This region, formed by the two cysteine residues that confer the cyclic conformation to the peptides, corresponds to the area of highest electronic hardness. For the Ptry6 peptide, the LUMO is similarly associated with Cys1, but in the N-terminal (Fig. 2b).

The three peptides exhibit the same distribution of HOMO and LUMO orbitals, notably Ptry6 being an open-side-chain peptide with the HOMO distributed on Cys6 residue and the LUMO on the other Cys1 residue. For Ptry9L and Ptry9D, the HOMO is distributed on the Cys9 residue without a stereocenter, and the LUMO is distributed on the disulfide bond closest to Lys3. Ptry6 displays the LUMO at the Cys1 residue and the HOMO at the Cys6 residue.

**Fig. 3:**
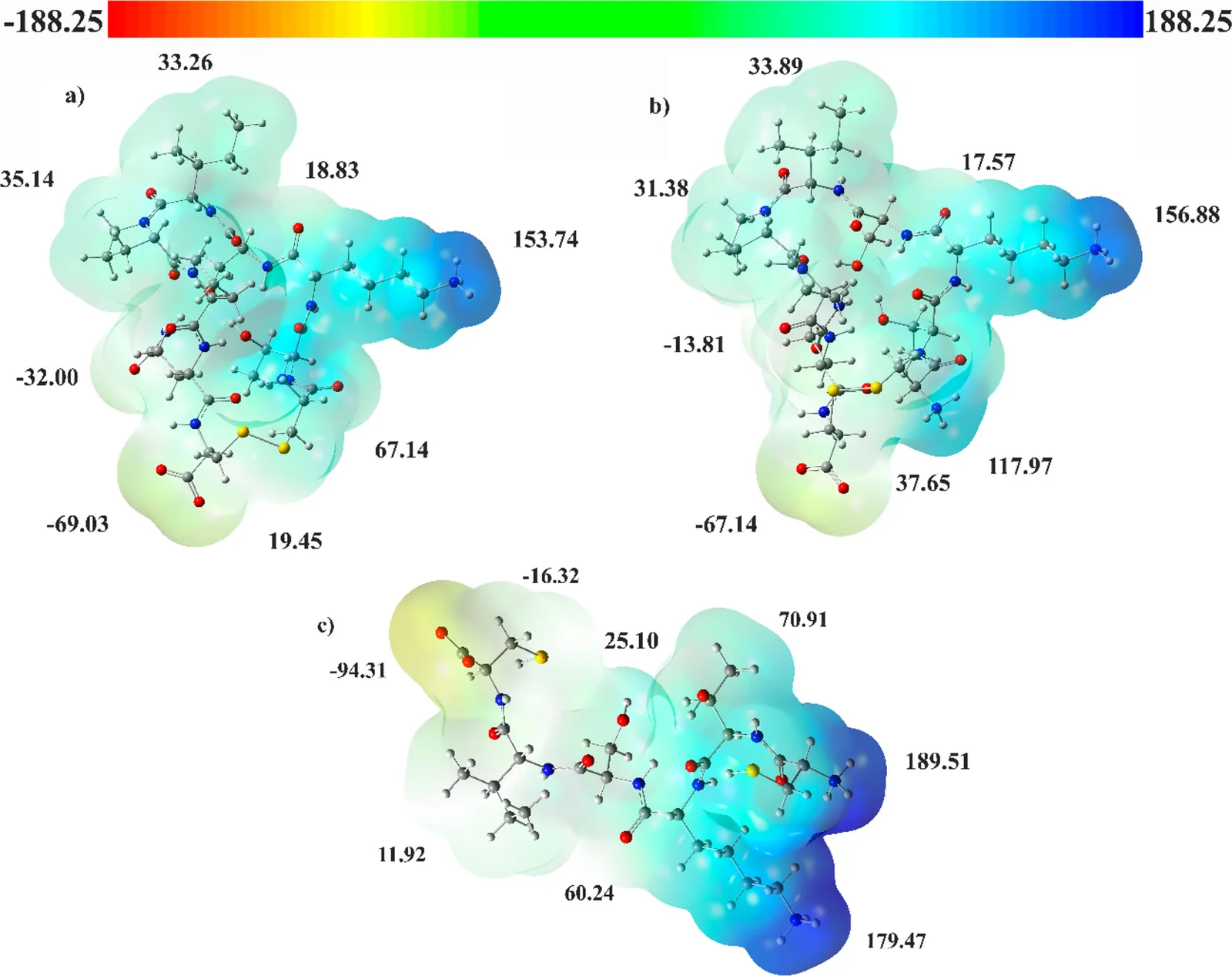
Molecular electrostatic potential map of **a**) Ptry9D, **b**) Ptry9L, and **c**) Ptry6 at B3LYP-D3/6–311+G(d,p) solvent model (values in kcal/mol)

The electrostatic potential map (Fig. 3) is a valuable tool for understanding changes in electronic density in charged regions resulting from conformational and chiral effects on the peptides. Both electron density functionals presented the same trend for the studied peptides. Therefore, the B3LYP-D3/6–311+G(d,p)-optimized structures were selected. Figure 3 exhibits regions of greater variation in electronic density at the C-terminal, N-terminal, and Lys3 regions for Ptry9D, Ptry9L, and Ptry6, respectively, suggesting a greater tendency and more favorable sites for interactions with trypsin-like proteins.

The crystallographic structures of 6EAT and 6E5M reveal a conserved interaction mechanism characterized by an electrostatic interaction between the Lys3 residue of the Ptry9L or Ptry9D peptides and the Asp189 residue of trypsin [29]. The Lys3 residue region of the Ptry9D, Ptry9L, and Ptry6 peptides (Fig. 3) exhibits electrostatic potential of 153.74 kcal/mol, 156.88 kcal/mol, and 179.47 kcal/mol, respectively. These values partially explain why Ptry9L exhibits a greater tendency to act as an acceptor than Ptry9D, showing a higher acceptor potential for nucleophilic interactions with the trypsin active site. The acceptor tendency of Ptry6 Lys3 is due to its open-chain conformation and low electronic density. The Lys3 residue in the BBI species has previously been identified as the key acceptor group in these ligands during interactions with trypsin or chymotrypsin, playing a crucial role in enzyme inhibition [4, 23].

The acceptor tendency at the N-terminus of Ptry9L (117.97 kcal/mol) is higher than that observed for Ptry9D (67.14 kcal/mol). In Ptry9D, the acceptor potential of the Cys1 region is reduced due to the steric hindrance effect of the N-terminal. The acceptor potentials of Lys3 and Cys1 are higher in Ptry9L than in Ptry9D. These results suggest a stronger inhibitory tendency for Ptry9L. The Ptry6 exhibits even higher acceptor potentials at Lys3 and Cys1, reaching 179.47 kcal/mol and 94.31 kcal/mol, respectively. However, intramolecular interactions within Ptry6 shorten the distance between the N-terminus and Lys3, potentially affecting the interaction at the enzyme’s active site. The disulfide bond region does not exhibit significant structural or charge differences between the Ptry9L and Ptry9D peptides. This region, where the LUMO orbital is located, presents charge values of 37.65 kcal/mol and 19.45 kcal/mol. Its electronic characteristics indicate a potential site for nucleophilic attack. The sulfur atom regions in Ptry6 correspond to the Cys6 and Cys1 residues, which present opposite charges of −16.32 kcal/mol and 70.91 kcal/mol. Therefore, the disulfide bond seems to stabilize the charge difference between sulfur regions, with an acceptor tendency.

Molecular electrostatic potential (MEP) profiles suggest that Ptry9L exhibits a greater tendency to interact with other proteins than Ptry9D. The electronegative regions of Ptry9D, Ptry9L, and Ptry6 (yellow to red colored regions) are localized at the C-terminal (COO^−^), specifically in the Cys9 and Cys6 regions. The charge at the C-terminal is higher in the Ptry6 peptide (−94.31 kcal/mol) than in the Ptry9L and Ptry9D peptides, −69.03 kcal/mol and −67.14 kcal/mol, respectively. The MEP analysis reveals that the Cys1 and Lys3 moieties of the Ptry9D and Ptry9L peptides are potential sites of nucleophilic attacks. These acceptor regions are oriented on the same side of the peptide, while the donor portion is located on the opposite side. Therefore, the MEP results indicate that the acceptor region of the molecule has a high potential for interaction with the catalytic site.

### IGMH studies

**Fig. 4:**
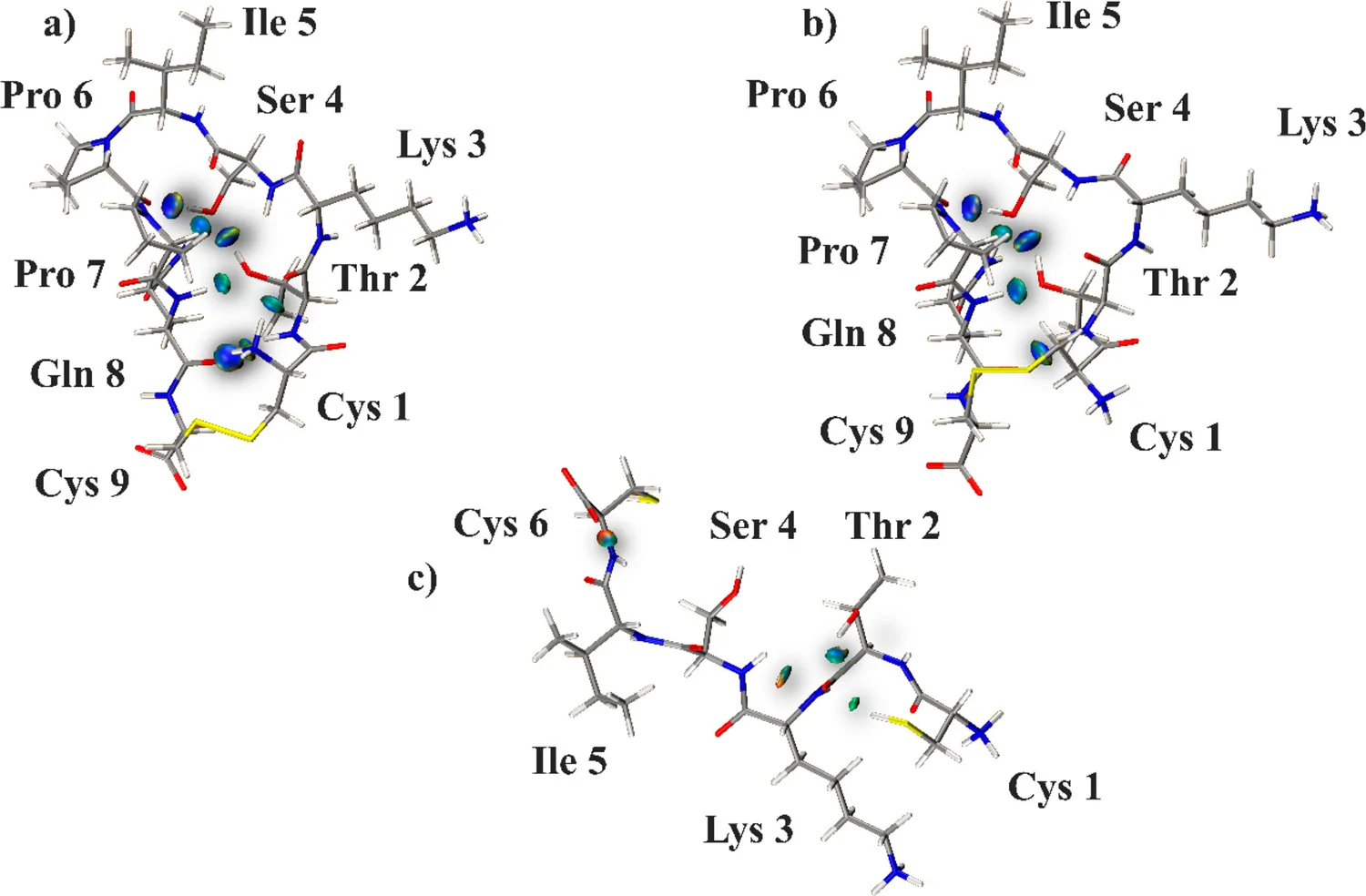
IGM isosurfaces of hydrogen bonds for Ptry9D (**a**), Ptry9L (**b**), and Ptry6 (**c**) at B3LYP-D3/6–311+D(d,p)-PCM calculation level

Intramolecular non-covalent hydrogen bonds and van der Waals interactions play a critical role in the structural stabilization of these peptides. Therefore, IGMH [42] was performed to analyze intramolecular non-covalent interactions in the peptide structures (Fig. 4). The IGMH study of the Ptry9D peptide reveals the presence of seven hydrogen bonds in the inner portion of Ptry9D, while five hydrogen bonds are present in the inner portion of Ptry9L and four in Ptry6. The localization of some of these hydrogen bonds differs between the Ptry9D and Ptry9L structures.

For the Ptry9D peptide (Fig. 4a), the three strongest interactions (navy color) are formed. The first hydrogen bond is formed between the carbonyl group of the backbone of Pro7 and the hydroxyl group of the side chain of Ser4. The second hydrogen bond was observed between the hydroxyl groups of the Thr2 and Ser4 side chains. The third hydrogen bond is located between the amine group of Gln8 and the Ser4 side chain. The other four interactions in Ptry9D occur between Gln8, Thr2, and Cys1. The first is located between the amine hydrogen of Gln8 and the oxygen atom of the hydroxyl group of the Thr2 side chain, and the second and third are between the carbonyl group of the backbone of Gln8 and the amine group of Thr2 and Cys1 residues, respectively. The last hydrogen bond is located between the amine hydrogen and the carbonyl group of the backbone of Thr2 (cyan).

The three strongest interactions were observed in Ptry9L (Fig. 4b, navy blue) and in Ptry9D (Pro7-Ser4, Gln8-Ser4, and Thr2-Ser4). Two less strong interactions are found in Ptry9L. One is between the Gln8 backbone amine group and the hydroxyl group of the Thr2 side chain. The second is the carbonyl group of the Gln8 backbone and the amine group of the Thr2 backbone. Therefore, Ptry9D presents a smaller structure than Ptry9L due to the greater number of internal hydrogen bonds, with a two-dimensional length of 18.31 Å (Ptry9L) and 17.81 Å (Ptry9D) (Table 2). The smaller conformation of Ptry9D brings the disulfide bond closer to sites with potential nucleophilic attack, specifically the N-terminal group.

The regions of the disulfide bond, between Cys1 and Cys9, and the C-terminal site do not present hydrogen bonds with the other residues. Therefore, the covalent interaction between sulfur atoms contributes to the stabilization of the cyclic peptide [11].

The Ptry6 exhibits four weaker van der Waals interactions (green color) compared to Ptry9D and Ptry9L, which are mainly between the amine and carbonyl groups of the backbone (Fig. 4c). The van der Waals interactions of Ptry6 occur between the amine and the carbonyl groups or side chain hydroxyl group of the following residues: Cys6 C-terminal, Ser4-Lys3, Thr2-Thr2 side chain, Thr2-Cys1 side chain.

The intramolecular hydrogen bond interactions of cyclic peptides, Ptry9D and Ptry9L, are shown in Fig. 4a and b (navy color disks) and van der Waals interactions of Ptry6 in Fig. 4c (green color disks). Considering that disulfide bonds confer stability in protein folding and that hydrogen bonds are energetically more stable than van der Waals interactions, cyclic peptides tend to be more stable than acyclic peptides. The lower stability of Ptry6 was confirmed by optimizing its structure in the absence and presence of an implicit solvent, resulting in changes in van der Waals interactions. However, the favorable conformation of Ptry6 for enzyme inhibition tends to mimic that presented for Ptry9L, with two potentially donor regions oriented toward trypsin and few interactions with neighboring residues [29].

### Molecular docking

**Fig. 5:**
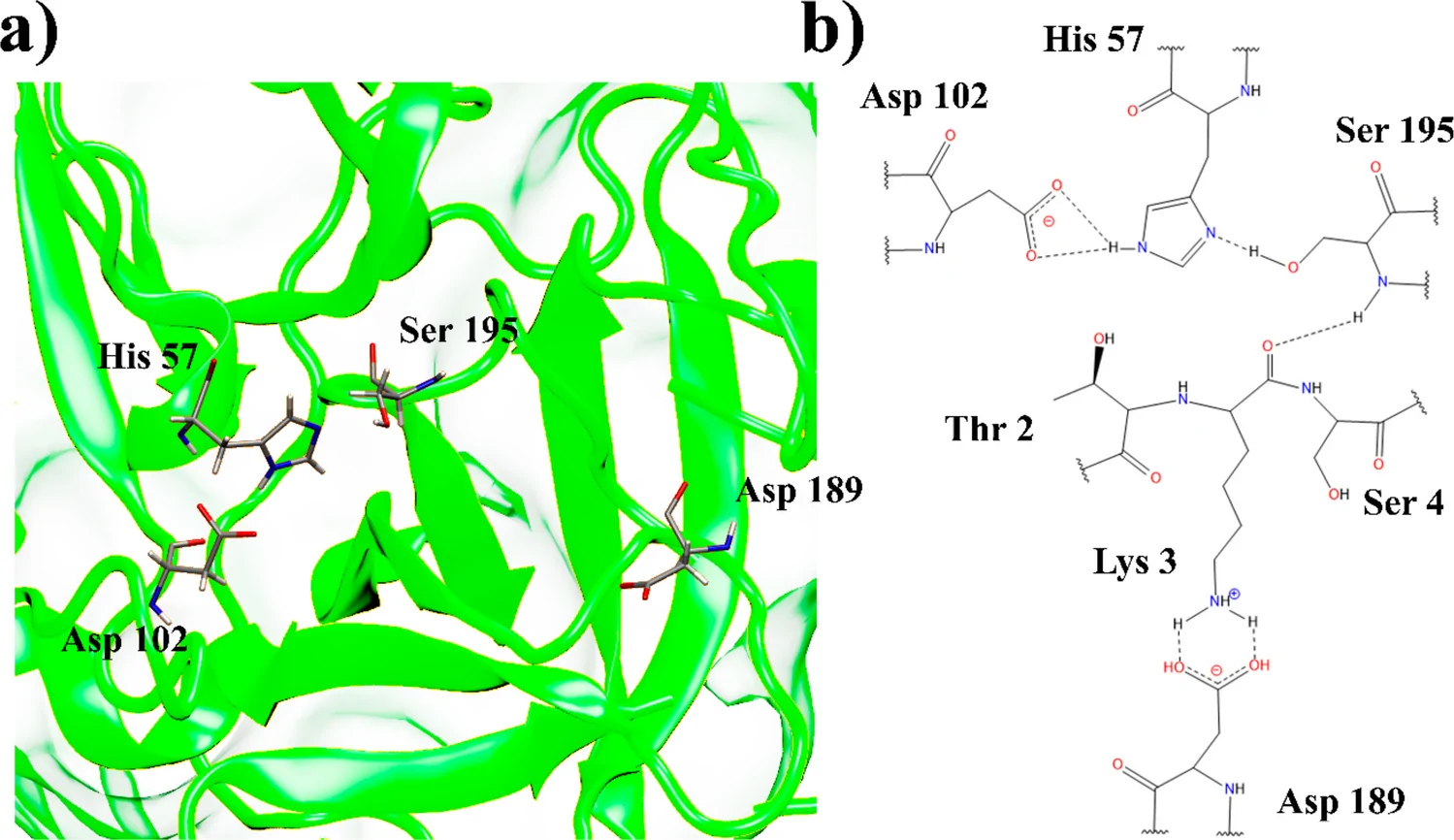
Crystallographic structure of Ptry9L (PDB code 6E5M). **a**) Highlighted catalytic triad residues His57, Asp102, and Ser195. S_1_ position is represented by Asp189, and **b**) 2D representation of the main molecular interactions of Ptry9L with trypsin residues involved in the inhibitory activity

Trypsin inhibition basically occurs in four phases: substrate binding, nucleophilic attack, protonation, and hydrolysis [29]. In the first stage, the P1 residue of the inhibitor interacts with the S1 catalytic pocket of the enzyme (Fig. 5), leading to nucleophilic attack by Ser195 and the proton transfer from Ser195 to His57. Prior to hydrolysis, Asp102 stabilizes His57, and a covalent bond is formed between the oxygen of the Ser195 side chain and the carbonyl group of the P1 residue of the inhibitor (Lys3). The negative charge on the carbonyl oxygen of the inhibitor is stabilized by hydrogen bonds with residues of the oxyanion hole [22, 26, 29].

In the enzyme-substrate mechanism, protonation state occurs when the His57 residue donates a proton to the nitrogen atom of the substrate’s amino group, allowing the substrate peptide bond to break and the free peptide to be released as a reaction product [29]. This interaction generates a tetrahedral covalent complex. The next step is the interaction of water molecules in the catalytic pocket, followed by hydrolysis. Finally, the interactions are broken, regenerating a new C-terminal and the Ser195 residue, with trypsin returning to its initial conformation [29].

Ptry9D and Ptry9L present a substrate-like mechanism for the recognition and inhibition of trypsin [24, 29]. The recognition process is driven by an electrostatic interaction between Lys3 in the peptides and Asp189 in trypsin (Fig. 5b) [29]. The cyclic conformation of these peptides allows a hydrogen bond between the carbonyl group of the Lys3 backbone and the amine group of the Ser195 backbone [29]. Consequently, the peptide fails to form a covalent bond with Ser195, thereby inhibiting trypsin’s catalytic activity [29]. The disulfide bond and the intermolecular hydrogen bonds shown in Fig. 4 uphold the cyclic structure and account for the inhibitory activity of those peptides. Table 3 presents the molecular docking results.

**Table 3:** Binding energy and structural and kinetic parameters observed from the best pose solution of the trypsin-peptide molecular docking study

Protein structure from PDB	Ligand	Geometry of the ligand in different moieties	Binding energy (score/kcal mol^−1^)	Half maximal inhibitory concentration (IC_50_/µM) [29]	Theoretical inhibition constant (*K*_i_/µM)	N hydrogen bonds
6EAT	Ptry9L	Solvent*	−5.82	50	45.01	7
		Vacuum**	−10.68		14.87	8
		PDB***	−13.52		0.0001232	10
	Ptry9D	solvent*	−6.13	50	32.04	7
		vacuum**	−5.75		61.12	6
		PDB***	−8.15		1.07	9
	Ptry6	Solvent*	1.20		0	4
		vacuum**	1.20		0	4
		PDB***	1.06		0	9

*Solvent is the quantum mechanics geometric structure of the ligand, optimized using the implicit solvent method (B3LYP/Def2-SVP/PCM)

**Vacuum—ligand structure optimized in gas phase (B3LYP/Def2-SVP)

***PDB—non-optimized ligand structure obtained from PDB structure

**Fig. 6:**
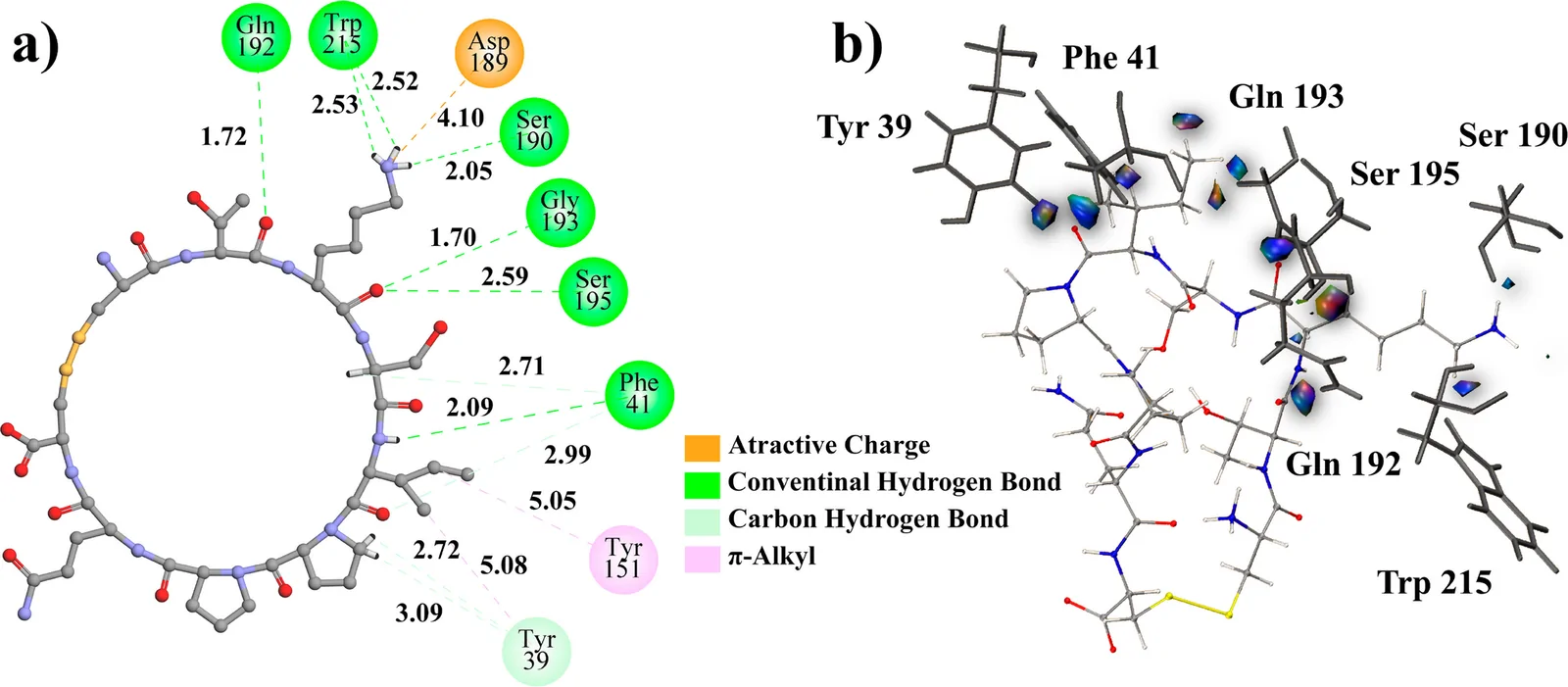
Molecular docking of the Ptry9D optimized structure with PCM implicit solvent B3LYP/Def2-SVP (complexed with 6E5M): **a**) Ptry9D with amino acids that perform interaction in 2D conformation and **b**) IGM studies of intermolecular hydrogen bonds

Molecular docking results indicate that Ptry9L and Ptry9D inhibit trypsin, whereas Ptry6 shows no inhibitory activity, in agreement with literature reports [29]. Both the PDB-derived structures and the vacuum-optimized models exhibit inhibitory activity in the order Ptry9L > Ptry9D. When optimized in the presence of solvent, the binding energy difference for the two peptides is less than 0.5 kcal/mol, indicating the same order of inhibitory activity. Furthermore, theoretical *K*ᵢ and IC₅₀ values of both solvent-optimized Ptry9L and Ptry9D are in agreement with the experimental IC₅₀ of ~ 50 µM [29]. Based on these results, subsequent structural analyses were performed only for the solvent-optimized Ptry9L and Ptry9D. The Ptry9D molecular docking study (Fig. 6a) shows 14 interactions with 9 enzyme residues, of which 7 are hydrogen bonds between Thr2 and Ile5. The Ser195 catalytic residue interacts through hydrogen bonds with the Ptry9D Lys3 backbone (2.59 Å). Ptry9D-Lys3 interacts electrostatically with residue Asp189 (4.10 Å) and through hydrogen bonds with residues Gly193 (1.70 Å), Ser190 (2.05 Å), and Trp215 (2.52 Å and 2.53 Å). Other hydrogen bonds were observed between Ptry9D-Phe41 and Gln192 (2.09 Å and 3.4 Å). All hydrogen bonds were confirmed by IGM studies, which suggest a strong tendency toward inhibition (Fig. 6b). A π-alkyl type interaction with Tyr151 was observed. Residues Cys1, Cys9, Gln8, Pro7, and Pro6 showed no interactions.

**Fig. 7:**
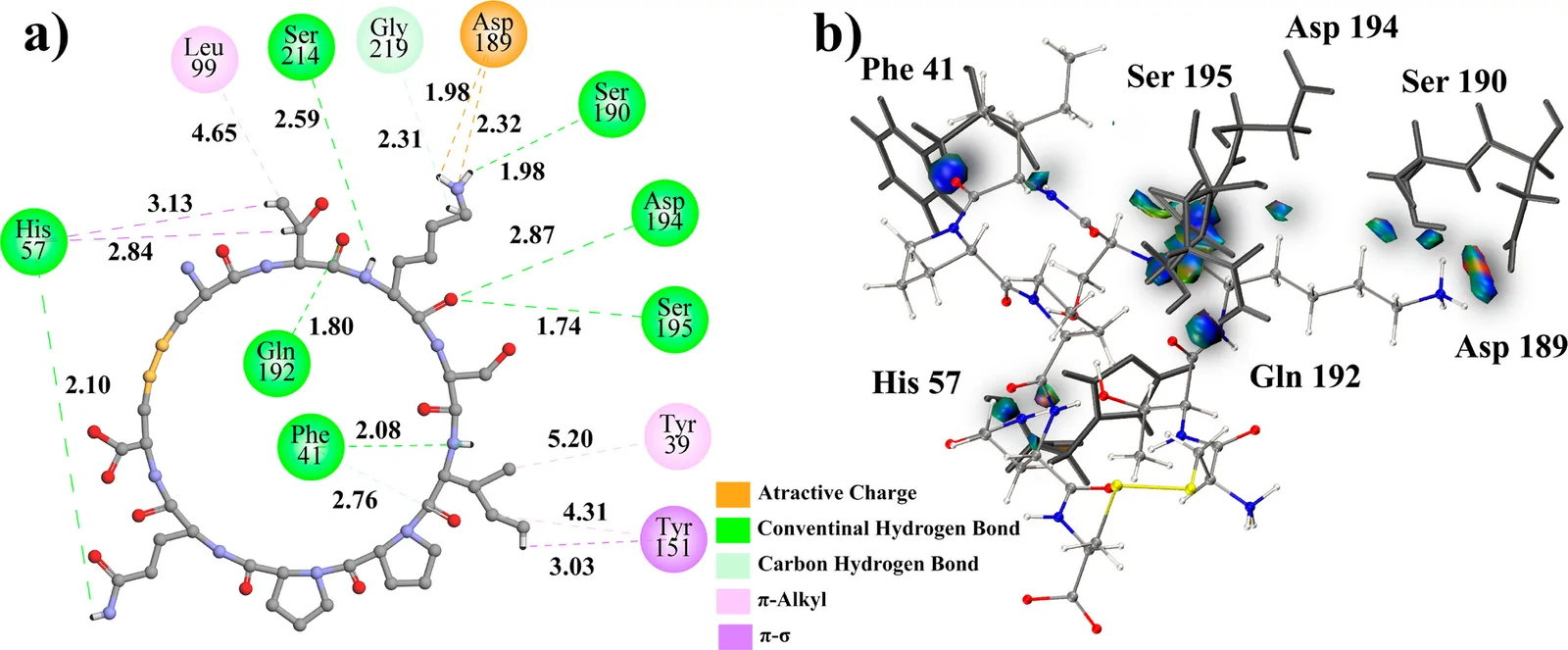
Molecular docking of the Ptry9L optimized structure with PCM implicitly solvent from B3LYP/Def2-SVP (complexed with 6E5M): **a**) Ptry9L with amino acids that perform interaction in 2D conformation. **b**) Main amino acids of Ptry9L involved in trypsin inhibition

The molecular docking study of Ptry9L reveals a greater number, 25, of interactions with 13 trypsin residues [29] compared to Ptry9D (eight hydrogen bonds) (Fig. 7). These interactions involve peptide residues Thr2 through Ile5 and Gln8. Lys3 forms hydrogen bonds with Ser190, Ser195, Gly193, Asp194, and Ser214 at distances of 2.46 Å, 1.74 Å, 2.35 Å, 2.87 Å, and 2.59 Å, respectively. A shorter electrostatic attraction was observed between Lys3 and Asp189 of trypsin (2.05 Å and 1.98 Å) compared to Ptry9D (4.1 Å), which is consistent with a strong hydrogen bond [4, 27, 29].

Ptry9L interacts with Ser190 and His57 of the catalytic site at distances of 2.46 Å and 2.10 Å, respectively. The hydrogen-bond and π-σ interactions between His57 of trypsin and Gln8 and Thr2 of Ptry9L indicate a higher affinity for Ptry9L than for Ptry9D. Additionally, hydrogen bonds between Thr2-Gln192 at 1.80 Å and Ile5-Phe41 at 2.08 Å, and hydrophobic interactions with Ile5-Tyr151 and Tyr39 (3.61 Å, 4.31 Å, and 5.20 Å) are shown. No interactions between His57 and Ser214 were observed in the IGM analysis (Fig. 7b), suggesting weak interactions despite their proximity in the molecular docking. All other interactions were confirmed by the IGM study.

Crystallographic and vacuum docking studies of the trypsin-peptide complexes resemble the solvent-based complex. The Thr2 to Ile5 residues of cyclic peptides constitute the main interaction region with trypsin in all complexes. The data obtained through comparative methods of electronic structure, electrostatic potential, molecular docking, and the Independent Gradient Model based on Hirshfeld partition of molecular density corroborate previously reported data and the interactions found in the crystallographic structures of Ptry9L and Ptry9D [29].

## Conclusions

Taken together, these results indicate that the solvent-optimized Ptry6 peptide presents considerable structural changes from its PDB structure, whereas Pty9L and Ptry9D show smaller structural changes. This difference in conformational changes is consistent with the cyclic structure of Ptry9D and Ptry9L, stabilized by the disulfide bond, which restricts conformational changes and preserves their inhibitory activity, making them potential subjects for further structural and biotechnological studies. The HOMO molecular orbital tends to be localized in the C-terminal region for all peptides, while the LUMO localization varies for cyclic and non-cyclic peptides. The Ptry6 LUMO orbital is localized in the Cys1 N-terminal, while in the Ptry9D and Ptry9L peptides, the LUMO is localized in the disulfide bond (Cys1 and Cys2), which is consistent with the cyclic characteristics of these molecules.

Non-cyclic peptides have potentially electropositive and electronegative N-terminal and C-terminal regions, respectively. The obtained DFT results indicated that the disulfide bond and the location of the LUMO orbital reduce the electronegative potential of the C-terminal region in both Ptry9L and Ptry9D. This electronic feature occurs without altering the acceptor potential of the N-terminal region, imparting an electropositive charge tendency to the cyclic peptides. Furthermore, the *Δ*_FMO_ reactivity indicates that the cyclic peptides are highly reactive and identify that the N-terminal region (Lys3) as the main acceptor of these BTCI-derived peptides. These results also indicate that cyclic peptides have an optimized sequence for inhibiting enzymes with high-density electron-catalytic sites, such as trypsin- and chymotrypsin-like enzymes.

The IGMH studies were relevant and corroborated previously reported data on the greater inhibitory activity related to the structural stability of the cyclic peptides Ptry9D and Ptry9L. The cyclic structure is maintained by a disulfide and internal hydrogen bonds between the Ser4, Pro7, Thr2, and Gln8 residues. The Ptry6 peptide exhibits only van der Waals interactions. The structural absence of the disulfide bond in Ptry6 was reflected in greater conformational freedom and lower electronic potentials at the lysine residue compared with the N-terminal of cyclic peptides, resulting in low scores in molecular docking studies. Due to these structural characteristics, Ptry6 did not exhibit inhibitory potential against trypsin, which is in agreement with the experimental data and corroborating its weak trypsin-inhibitory profile. Molecular docking studies demonstrated that Ptry9L exhibits a greater tendency to inhibit trypsin than Ptry9D, consistent with a lower electron density revealed by the electrostatic potential map. These results highlight that the stereoisomerism of L-Cys1 is associated with a greater number of internal hydrogen bonds, conferring a lower electronic potential density in Ptry9L. In these molecular docking studies, the residues Thr2, Lys3, Ser4, and Ile5 were highlighted, forming hydrogen bonds with trypsin residues: Asp189, Ser190, Gln192, Gly193, and Ser195. These interactions are essential for inhibiting trypsin catalytic activity, as previously described. Therefore, due to its optimal geometric conformation, physical, and electronic properties, the Ptry9L peptide is a more effective inhibitor than Ptry9D. Finally, this study demonstrated that the disulfide bond confers structural stability by restricting the flexibility of these peptides and that the L-stereoisomer configuration increases the electronic potential at the key Lys3 residue.

## Supplementary information

Below is the link to the electronic supplementary material.

Supplementary file1 (DOCX 1915 kb)

## Data Availability

The authors declare that the data supporting the findings of this study are available within the paper and its Supplementary Information file. Should any raw data files be needed in another format they are available from the corresponding author upon reasonable request.
