# Supplementary material for: Molecular modeling and quantum chemical calculation study of derived peptides from a Bowman-Birk inhibitor in complexes with trypsin
Source: J Mol Model. 2026 Sep 5;32(10):348. doi: 10.1007/s00894-026-06931-0 (PMC13546364; doi:10.1007/s00894-026-06931-0)
Supplement: Supplementary file 1 [file 894_2026_6931_MOESM1_ESM.docx]

**Molecular modeling and quantum chemical calculation study of derived peptides from a Bowman-Birk inhibitor in complexes with trypsin.**

Luiz. F. M. A. Benício^1^, Érica. C. M. Nascimento^2^, Sonia M. de Freitas^3^, João. B. L. Martins^1,2^

1. University of Brasília, Department of Pharmacy, Faculty of Health Sciences, Brasília, DF 70910-900, Brazil.

2. University of Brasilia, Institute of Chemistry, Computational Chemistry Laboratory, Brasilia, DF, Brazil, 70910-900

3. University of Brasilia, Institute of Biological Science, Biophysics Laboratory, Brasilia, DF, Brazil, 70910-900.

**Supplementary information**

Table S1 – RMSD values (Å) between the crystallographic structure and geometries of the obtained peptides using the B3LYP/Def2-SVP and B3LYP/6-311+G(d,p) in vacuum and implicit solvent PCM methods.

|  | Conformation | Rotatable bonds | Def2-SVP | | 6-311+G(d,p) | |
| --- | --- | --- | --- | --- | --- | --- |
|  |  |  | vacuum | solvent | vacuum | solvent |
| Ptry9D | Cyclic | 17 | 1.33 | 1.18 | 0.76 | 0.95 |
| Ptry9L | Cyclic | 17 | 0.65 | 0.64 | 0.69 | 0.76 |
| Ptry6 | Noncyclic | 32 | 3.27 | 3.11 | 4.03 | 3.37 |

Table S2. Calculated molecular size (Å) and gap energies (kcal/mol) at B3LYP/Def2-SVP and B3LYP/6-311+G(d,p) level using PCM implicit solvent model for Ptry9D, Ptry9L, and Ptry6 molecules.

|  | **Def2-SVP solvent** | | **6-311+G(d,p) solvent** | |
| --- | --- | --- | --- | --- |
|  | *Size* | *Gap*  *(HOMO-LUMO)* | *Size* | *Gap*  *(HOMO-LUMO)* |
| Ptry9D | 18.22 | 78.08 | 18.40 | 125.68 |
| Ptry9L | 18.15 | 74.05 | 18.38 | 120.37 |
| Ptry6 | 18.32 | 89.39 | 18.78 | 138.59 |

Table S3. Calculated molecular properties at B3LYP-D3/Def2-SVP and B3LYP-D3/6-311+G(d,p) level using PCM implicit solvent model for Ptry9D, Ptry9L, and Ptry6 molecules.

|  | ***Def2-SVP solvent*** | | | | | | | |
| --- | --- | --- | --- | --- | --- | --- | --- | --- |
|  | *Size* | *HOMO* | *LUMO* | *Gap* | *χ* | *η* | *ω* | *μ* |
|  |  |  |  | *(HOMO-LUMO)* |  |  |  |  |
| *Ptry9D* | 17.96 | -138.90 | -21.45 | 117.44 | 3.48 | 2.55 | 2.37 | -3.48 |
| *Ptry9L* | 18.01 | -134.04 | -25.61 | 108.43 | 3.46 | 2.35 | 2.55 | -3.46 |
| *Ptry6* | 17.80 | -129.07 | -13.58 | 115.49 | 3.09 | 2.50 | 1.91 | -3.09 |

|  | ***6-311+G(d,p) solvent*** | | | | | | | |
| --- | --- | --- | --- | --- | --- | --- | --- | --- |
|  | *Size* | *HOMO* | *LUMO* | *Gap* | *χ* | *η* | *ω* | *μ* |
|  |  |  |  | *(HOMO-LUMO)* |  |  |  |  |
| *Ptry9D* | 17.81 | -152.36 | -32.42 | 119.95 | 4.01 | 2.60 | 3.09 | -4.01 |
| *Ptry9L* | 18.31 | -149.36 | -35.18 | 114.19 | 4.00 | 2.48 | 3.23 | -4.00 |
| *Ptry6* | 19.03 | -146.32 | -24.34 | 121.98 | 3.70 | 2.64 | 2.59 | -3.70 |

Figure S1. Optimized structures of peptides derived from BTCI using B3LYP/Def2-SVP. a) Ptry9D, with Cys1 structure represented by Cys D highlighted; b) Ptry9L, with zoom in Cys1 structure represented by Cys L highlighted; and **c**) Ptry6 structure.

HOMO LUMO

Figure S2. Molecular orbitals HOMO and LUMO of (a and b) Ptry9D, (c and d) Ptry9L and (e and f) Ptry6 for the B3LYP/Def2-SVP in solvent PCM model. Atom color code: navy (N), gray (C), red (O), yellow (S), and white (H). Red and green colors are used for the orbital phases.

Figure S3. Electrostatic potential map of (a) Ptry9D, (b) Ptry9L and (c) Ptry6 at B3LYP/Def2-SVP-PCM solvent model (values in kcal/mol).

Figure S4. IGM isosurfaces of hydrogen bonds for Ptry9D (a), Ptry9L (b), and (c) Ptry6, at B3LYP/Def2-SVP-PCM calculation level.

Table S4. Cartesian coordinates of Ptry6 optimized at B3LYP-D3/6-311+G(d,p) and B3LYP-D3/Def2-SVP

|  | B3LYP-D3/6-311+G(d,p) | | | B3LYP-D3/Def2-SVP | | | |
| --- | --- | --- | --- | --- | --- | --- | --- |
| Symbol | X | Y | Z | Symbol | X | Y | Z |
| N | 0 | 0 | 0 | N | 0 | 0 | 0 |
| C | 1.5086 | 0 | 0 | C | 1.4996 | 0 | 0 |
| C | 1.9123 | 1.4827 | 0 | C | 1.8861 | 1.4914 | 0 |
| O | 1.1643 | 2.3095 | -0.5202 | O | 1.0613 | 2.3202 | -0.38 |
| C | 2.0201 | -0.7096 | -1.2698 | C | 2.0363 | -0.7086 | -1.2614 |
| S | 3.8337 | -0.98 | -1.2903 | S | 3.8266 | -1.078 | -1.1923 |
| H | -0.3949 | -0.8598 | -0.3865 | H | -0.4157 | -0.7908 | -0.5061 |
| H | -0.3757 | 0.1164 | 0.9441 | H | -0.3845 | -0.0014 | 0.9529 |
| H | 1.8411 | -0.5254 | 0.8931 | H | 1.8468 | -0.5209 | 0.9012 |
| H | 1.5899 | -1.7113 | -1.3146 | H | 1.5427 | -1.6863 | -1.3619 |
| H | 1.7168 | -0.1645 | -2.1642 | H | 1.8197 | -0.1155 | -2.1615 |
| H | 4.2051 | 0.2804 | -1.6085 | H | 4.2685 | 0.1281 | -1.628 |
| N | 3.1131 | 1.7687 | 0.5182 | N | 3.1465 | 1.7786 | 0.3506 |
| C | 3.7379 | 3.0704 | 0.3115 | C | 3.7602 | 3.0265 | -0.0573 |
| C | 4.1555 | 3.1931 | -1.1637 | C | 3.662 | 3.1586 | -1.5864 |
| O | 4.9615 | 2.3926 | -1.6462 | O | 3.604 | 2.1713 | -2.3139 |
| C | 4.9884 | 3.1887 | 1.217 | C | 5.2511 | 2.9984 | 0.4104 |
| O | 5.8205 | 2.0355 | 1.0611 | O | 5.8275 | 1.7491 | 0.1053 |
| C | 4.6237 | 3.3013 | 2.6882 | C | 5.3549 | 3.2182 | 1.9152 |
| H | 3.7176 | 1.0216 | 0.8408 | H | 3.8066 | 1.0132 | 0.4947 |
| H | 3.0252 | 3.8519 | 0.5776 | H | 3.2428 | 3.877 | 0.4117 |
| H | 5.5297 | 4.0898 | 0.9032 | H | 5.7832 | 3.8133 | -0.1079 |
| H | 6.0159 | 1.9369 | 0.1161 | H | 6.1851 | 1.7248 | -0.8085 |
| H | 5.5352 | 3.3703 | 3.2843 | H | 6.4117 | 3.177 | 2.2165 |
| H | 4.0228 | 4.1958 | 2.8645 | H | 4.9401 | 4.1958 | 2.2025 |
| H | 4.0584 | 2.4265 | 3.0179 | H | 4.809 | 2.4324 | 2.4602 |
| N | 3.6144 | 4.2186 | -1.8493 | N | 3.711 | 4.4188 | -2.0823 |
| C | 3.8355 | 4.4358 | -3.2763 | C | 3.7067 | 4.658 | -3.5218 |
| C | 4.6431 | 5.7095 | -3.5781 | C | 4.9543 | 4.0678 | -4.2072 |
| O | 4.8557 | 6.0395 | -4.7426 | O | 4.924 | 3.7377 | -5.3873 |
| C | 2.5069 | 4.4159 | -4.0472 | C | 3.5349 | 6.1436 | -3.8519 |
| C | 1.8021 | 3.0594 | -3.9585 | C | 2.1669 | 6.7005 | -3.4492 |
| C | 0.4825 | 3.0493 | -4.7377 | C | 2.0095 | 8.1782 | -3.8185 |
| C | -0.2061 | 1.6974 | -4.6173 | C | 0.6416 | 8.7056 | -3.4107 |
| N | -1.4864 | 1.6655 | -5.4216 | N | 0.4695 | 10.1505 | -3.7894 |
| H | 2.8643 | 4.7339 | -1.4081 | H | 3.722 | 5.2097 | -1.4461 |
| H | 4.4638 | 3.6107 | -3.6206 | H | 2.875 | 4.0894 | -3.9677 |
| H | 1.8541 | 5.2075 | -3.6599 | H | 4.3444 | 6.7213 | -3.3725 |
| H | 2.7254 | 4.6638 | -5.0877 | H | 3.6721 | 6.2506 | -4.9394 |
| H | 2.4657 | 2.2814 | -4.3528 | H | 1.3773 | 6.1083 | -3.9433 |
| H | 1.6052 | 2.8089 | -2.9123 | H | 2.0093 | 6.5772 | -2.3642 |
| H | -0.1783 | 3.8345 | -4.3544 | H | 2.7987 | 8.771 | -3.3242 |
| H | 0.6746 | 3.2713 | -5.7929 | H | 2.1465 | 8.3036 | -4.9064 |
| H | 0.4165 | 0.8894 | -5.0001 | H | -0.1699 | 8.1542 | -3.9043 |
| H | -0.4835 | 1.4723 | -3.5881 | H | 0.49 | 8.6458 | -2.3244 |
| H | -1.9474 | 0.7552 | -5.3576 | H | -0.4517 | 10.5116 | -3.5086 |
| H | -2.1526 | 2.3698 | -5.0948 | H | 1.1765 | 10.7516 | -3.3441 |
| N | 5.0884 | 6.4102 | -2.5142 | N | 6.0469 | 3.938 | -3.4188 |
| C | 6.027 | 7.5014 | -2.6579 | C | 7.1247 | 3.007 | -3.7145 |
| C | 7.4552 | 6.9609 | -2.9189 | C | 6.5639 | 1.5782 | -3.5577 |
| O | 7.7528 | 5.7889 | -2.7159 | O | 6.5451 | 0.994 | -2.4665 |
| C | 5.9844 | 8.3763 | -1.4045 | C | 8.2997 | 3.2847 | -2.7792 |
| O | 6.3076 | 7.5435 | -0.2881 | O | 7.7918 | 3.4431 | -1.4665 |
| H | 5.016 | 5.9957 | -1.5959 | H | 5.971 | 4.1964 | -2.4404 |
| H | 5.7239 | 8.1086 | -3.5139 | H | 7.4567 | 3.1546 | -4.7527 |
| H | 6.6995 | 9.1993 | -1.5007 | H | 9.0174 | 2.4476 | -2.8477 |
| H | 4.9781 | 8.7958 | -1.3013 | H | 8.8086 | 4.2033 | -3.1229 |
| H | 6.2344 | 8.0628 | 0.5196 | H | 8.4995 | 3.741 | -0.8803 |
| N | 8.3184 | 7.895 | -3.3729 | N | 6.0413 | 1.0503 | -4.6725 |
| C | 9.7524 | 7.6745 | -3.5344 | C | 5.3841 | -0.2467 | -4.6808 |
| C | 10.4553 | 8.6632 | -2.5907 | C | 6.4443 | -1.332 | -4.4005 |
| O | 10.2395 | 9.8734 | -2.6983 | O | 7.534 | -1.3055 | -4.9675 |
| C | 10.2128 | 7.8828 | -4.9952 | C | 4.6681 | -0.5074 | -6.0252 |
| C | 9.4143 | 7.0126 | -5.9868 | C | 3.7403 | 0.6565 | -6.4446 |
| C | 11.729 | 7.6669 | -5.1113 | C | 3.9103 | -1.8422 | -5.9755 |
| C | 9.5418 | 5.496 | -5.7944 | C | 2.6524 | 1.0326 | -5.4351 |
| H | 8.0016 | 8.8471 | -3.4959 | H | 6.0274 | 1.6212 | -5.5106 |
| H | 9.9466 | 6.6477 | -3.226 | H | 4.6405 | -0.2541 | -3.8705 |
| H | 10.0012 | 8.9305 | -5.2401 | H | 5.4625 | -0.5909 | -6.789 |
| H | 8.358 | 7.2922 | -5.9278 | H | 4.336 | 1.5594 | -6.6566 |
| H | 9.7458 | 7.28 | -6.9964 | H | 3.2783 | 0.3744 | -7.4066 |
| H | 12.049 | 7.7773 | -6.1505 | H | 3.4519 | -2.0549 | -6.9538 |
| H | 12.285 | 8.3928 | -4.5126 | H | 4.5542 | -2.6901 | -5.7014 |
| H | 12.0204 | 6.6681 | -4.7741 | H | 3.1018 | -1.8098 | -5.2269 |
| H | 8.9655 | 4.9688 | -6.5597 | H | 2.0177 | 1.8368 | -5.8408 |
| H | 10.5793 | 5.1611 | -5.8768 | H | 1.9928 | 0.1818 | -5.1995 |
| H | 9.1578 | 5.1836 | -4.8203 | H | 3.0818 | 1.4025 | -4.4928 |
| N | 11.2653 | 8.1118 | -1.672 | N | 6.0608 | -2.2702 | -3.5162 |
| C | 12.0854 | 8.8625 | -0.7366 | C | 6.8494 | -3.416 | -3.1221 |
| C | 13.5247 | 8.2512 | -0.6697 | C | 6.063 | -4.762 | -3.3348 |
| O | 14.4028 | 8.9983 | -0.1775 | O | 6.6722 | -5.7883 | -2.9613 |
| C | 11.5028 | 8.8435 | 0.6864 | C | 7.283 | -3.3033 | -1.6553 |
| S | 9.7961 | 9.5211 | 0.8573 | S | 8.3339 | -1.8453 | -1.2564 |
| O | 13.6512 | 7.0685 | -1.0741 | O | 4.9193 | -4.6727 | -3.8377 |
| H | 11.5002 | 7.1292 | -1.7391 | H | 5.0979 | -2.2705 | -3.1927 |
| H | 12.153 | 9.8917 | -1.0885 | H | 7.7452 | -3.4351 | -3.7602 |
| H | 11.4218 | 7.8137 | 1.0419 | H | 6.395 | -3.2914 | -1.0024 |
| H | 12.1713 | 9.3844 | 1.3548 | H | 7.8744 | -4.1924 | -1.3991 |
| H | 10.07 | 10.7676 | 0.4232 | H | 7.4784 | -0.8961 | -1.7099 |
| H | -0.3199 | 0.8046 | -0.5586 | H | -0.2856 | 0.8996 | -0.4424 |
| H | -1.3091 | 1.8483 | -6.4126 | H | 0.5525 | 10.2932 | -4.8051 |

Table S5. Cartesian coordinates of Ptry9D optimized at B3LYP-D3/6-311+G(d,p) and B3LYP-D3/Def2-SVP

|  | B3LYP-D3/6-311+G(d,p) | | | B3LYP-D3/Def2-SVP | | | |
| --- | --- | --- | --- | --- | --- | --- | --- |
| Symbol | X | Y | Z | Symbol | X | Y | Z |
| N | 0 | 0 | 0 | N | 0 | 0 | 0 |
| C | 1.5146 | 0 | 0 | C | 1.5067 | 0 | 0 |
| C | 1.9713 | 1.4701 | 0 | C | 1.9564 | 1.473 | 0 |
| O | 2.7782 | 1.862 | 0.8307 | O | 2.7285 | 1.8857 | 0.8497 |
| C | 2.103 | -0.8028 | -1.1575 | C | 2.0991 | -0.8044 | -1.1552 |
| S | 1.6977 | -2.6014 | -1.1506 | S | 1.7156 | -2.6062 | -1.1419 |
| H | -0.3731 | -0.8577 | 0.4071 | H | -0.3837 | -0.8625 | 0.3986 |
| H | -0.358 | 0.089 | -0.9714 | H | -0.352 | 0.0944 | -0.9847 |
| H | 1.8291 | -0.4161 | 0.9548 | H | 1.8337 | -0.4124 | 0.962 |
| H | 1.8266 | -0.374 | -2.1161 | H | 1.8149 | -0.3755 | -2.1223 |
| H | 3.1908 | -0.7762 | -1.0643 | H | 3.1957 | -0.7661 | -1.0644 |
| N | 1.3639 | 2.2793 | -0.9062 | N | 1.3615 | 2.2704 | -0.9316 |
| C | 1.1417 | 3.665 | -0.5427 | C | 1.1295 | 3.6492 | -0.5628 |
| C | 0.2302 | 3.6989 | 0.7147 | C | 0.1643 | 3.6684 | 0.6522 |
| O | -0.3716 | 2.7013 | 1.1139 | O | -0.4735 | 2.6721 | 0.996 |
| C | 0.4537 | 4.4548 | -1.6744 | C | 0.4947 | 4.4791 | -1.699 |
| O | -0.9083 | 4.0001 | -1.7214 | O | -0.8793 | 4.0993 | -1.7679 |
| C | 1.1488 | 4.3161 | -3.0229 | C | 1.2007 | 4.3258 | -3.0396 |
| H | 0.6998 | 1.8722 | -1.5618 | H | 0.697 | 1.8521 | -1.5887 |
| H | 2.0981 | 4.1377 | -0.3118 | H | 2.0854 | 4.1106 | -0.2747 |
| H | 0.4642 | 5.5096 | -1.3808 | H | 0.557 | 5.5374 | -1.3905 |
| H | -1.5003 | 4.6863 | -2.0882 | H | -1.4334 | 4.7929 | -2.1955 |
| H | 0.629 | 4.9269 | -3.7641 | H | 0.6835 | 4.9356 | -3.7957 |
| H | 2.1839 | 4.6599 | -2.9552 | H | 2.2454 | 4.6644 | -2.9687 |
| H | 1.148 | 3.2802 | -3.3658 | H | 1.1962 | 3.279 | -3.3754 |
| N | 0.1152 | 4.9159 | 1.2788 | N | 0.0346 | 4.8724 | 1.2419 |
| C | -0.8283 | 5.2618 | 2.3366 | C | -0.9968 | 5.2145 | 2.2099 |
| C | -1.9081 | 6.2381 | 1.8205 | C | -1.9162 | 6.3194 | 1.6471 |
| O | -2.507 | 6.9898 | 2.5819 | O | -2.4631 | 7.1409 | 2.3689 |
| C | -0.1121 | 5.8056 | 3.5766 | C | -0.4172 | 5.5753 | 3.578 |
| C | 0.8258 | 4.7754 | 4.2099 | C | 0.317 | 4.4004 | 4.2248 |
| C | 1.5238 | 5.3343 | 5.4545 | C | 0.9089 | 4.7573 | 5.5908 |
| C | 2.4652 | 4.3068 | 6.0657 | C | 1.6013 | 3.5578 | 6.2212 |
| N | 3.132 | 4.8581 | 7.3063 | N | 2.1892 | 3.8992 | 7.5621 |
| H | 0.7064 | 5.6605 | 0.9341 | H | 0.6541 | 5.6216 | 0.948 |
| H | -1.3544 | 4.3381 | 2.5944 | H | -1.6286 | 4.3163 | 2.31 |
| H | 0.4483 | 6.7062 | 3.3005 | H | 0.2625 | 6.4384 | 3.4679 |
| H | -0.874 | 6.1169 | 4.2936 | H | -1.2471 | 5.9129 | 4.2163 |
| H | 0.254 | 3.8808 | 4.481 | H | -0.3801 | 3.5513 | 4.3341 |
| H | 1.5781 | 4.4595 | 3.4799 | H | 1.1227 | 4.0523 | 3.5558 |
| H | 2.0872 | 6.2347 | 5.1871 | H | 1.6301 | 5.5854 | 5.479 |
| H | 0.7727 | 5.6294 | 6.195 | H | 0.1083 | 5.1171 | 6.2598 |
| H | 1.9377 | 3.405 | 6.3752 | H | 0.9028 | 2.7267 | 6.3887 |
| H | 3.2673 | 4.0302 | 5.3823 | H | 2.4292 | 3.1936 | 5.5978 |
| H | 3.7684 | 4.177 | 7.7267 | H | 2.6608 | 3.0917 | 7.9899 |
| H | 2.4435 | 5.1136 | 8.0187 | H | 1.47 | 4.2149 | 8.2269 |
| N | -2.1461 | 6.1947 | 0.4868 | N | -2.0725 | 6.2942 | 0.2964 |
| C | -3.2629 | 6.9026 | -0.0985 | C | -3.0829 | 7.0952 | -0.3485 |
| C | -4.5975 | 6.2379 | 0.3062 | C | -4.4903 | 6.5644 | 0.0137 |
| O | -4.6351 | 5.1161 | 0.7976 | O | -4.6419 | 5.5087 | 0.6159 |
| C | -3.1023 | 6.9736 | -1.6271 | C | -2.8342 | 7.1452 | -1.8658 |
| O | -2.9945 | 5.6841 | -2.2373 | O | -2.7992 | 5.8563 | -2.4633 |
| H | -1.7682 | 5.4189 | -0.0406 | H | -1.7416 | 5.4778 | -0.211 |
| H | -3.2624 | 7.9278 | 0.2815 | H | -3.0155 | 8.1258 | 0.0358 |
| H | -3.936 | 7.5293 | -2.0636 | H | -3.5968 | 7.7761 | -2.3507 |
| H | -2.1772 | 7.5026 | -1.8585 | H | -1.8525 | 7.6087 | -2.0429 |
| H | -3.8521 | 5.1959 | -2.1843 | H | -3.7079 | 5.4394 | -2.4662 |
| N | -5.6938 | 6.9831 | 0.0411 | N | -5.5148 | 7.3489 | -0.3938 |
| C | -7.0561 | 6.4617 | 0.12 | C | -6.9134 | 6.954 | -0.2915 |
| C | -7.6234 | 6.5602 | -1.3074 | C | -7.465 | 6.9796 | -1.729 |
| O | -7.8068 | 7.6768 | -1.8029 | O | -7.6028 | 8.0619 | -2.297 |
| C | -7.9342 | 7.2283 | 1.1317 | C | -7.7311 | 7.8744 | 0.6405 |
| C | -7.3094 | 7.1625 | 2.5365 | C | -7.1063 | 7.9089 | 2.0462 |
| C | -9.3627 | 6.6657 | 1.106 | C | -9.1971 | 7.4244 | 0.6618 |
| C | -7.9866 | 8.0784 | 3.561 | C | -7.7172 | 8.9536 | 2.982 |
| H | -5.5881 | 7.9015 | -0.368 | H | -5.3282 | 8.2188 | -0.8832 |
| H | -6.9635 | 5.4319 | 0.4569 | H | -6.9121 | 5.9465 | 0.1389 |
| H | -7.9624 | 8.2754 | 0.8075 | H | -7.6829 | 8.8891 | 0.2058 |
| H | -7.3399 | 6.124 | 2.8891 | H | -7.1907 | 6.9033 | 2.4976 |
| H | -6.2522 | 7.4344 | 2.4665 | H | -6.0265 | 8.1075 | 1.9495 |
| H | -10.0139 | 7.2179 | 1.7854 | H | -9.8079 | 8.0846 | 1.2943 |
| H | -9.8051 | 6.7283 | 0.1087 | H | -9.6418 | 7.4366 | -0.3452 |
| H | -9.3693 | 5.6151 | 1.4158 | H | -9.2852 | 6.3998 | 1.0619 |
| H | -7.4603 | 8.0415 | 4.5188 | H | -7.1833 | 8.9782 | 3.9452 |
| H | -7.9821 | 9.1179 | 3.2174 | H | -7.6569 | 9.9624 | 2.5399 |
| H | -9.0249 | 7.7922 | 3.7459 | H | -8.7773 | 8.749 | 3.1996 |
| N | -7.8705 | 5.4242 | -1.9872 | N | -7.7479 | 5.8039 | -2.3373 |
| C | -7.609 | 4.0569 | -1.5297 | C | -7.4744 | 4.4678 | -1.8128 |
| C | -6.1041 | 3.7483 | -1.5279 | C | -5.9665 | 4.1899 | -1.7657 |
| O | -5.3366 | 4.3861 | -2.2719 | O | -5.2055 | 4.8047 | -2.5364 |
| C | -8.3287 | 3.1955 | -2.5933 | C | -8.1316 | 3.538 | -2.86 |
| C | -8.1768 | 4.0253 | -3.876 | C | -7.9512 | 4.318 | -4.1691 |
| C | -8.3276 | 5.471 | -3.3914 | C | -8.1616 | 5.7764 | -3.7499 |
| H | -8.0362 | 3.8948 | -0.5423 | H | -7.9271 | 4.3334 | -0.8228 |
| H | -7.9024 | 2.1952 | -2.6715 | H | -7.664 | 2.5433 | -2.8759 |
| H | -9.3806 | 3.1004 | -2.314 | H | -9.1978 | 3.4171 | -2.614 |
| H | -7.1825 | 3.8765 | -4.3016 | H | -6.9261 | 4.1816 | -4.5446 |
| H | -8.9173 | 3.7651 | -4.6324 | H | -8.6512 | 4.0009 | -4.9546 |
| H | -7.7224 | 6.1823 | -3.9539 | H | -7.5622 | 6.4924 | -4.3299 |
| H | -9.3672 | 5.8103 | -3.4205 | H | -9.2181 | 6.0842 | -3.8318 |
| N | -5.6873 | 2.7489 | -0.7489 | N | -5.5413 | 3.2417 | -0.9304 |
| C | -4.2806 | 2.3382 | -0.7577 | C | -4.1416 | 2.8292 | -0.9328 |
| C | -3.9668 | 1.6302 | -2.0846 | C | -3.8893 | 1.9694 | -2.1847 |
| O | -4.8154 | 0.9607 | -2.6702 | O | -4.7989 | 1.3612 | -2.7401 |
| C | -4.1804 | 1.3802 | 0.4459 | C | -4.0035 | 2.0378 | 0.3788 |
| C | -5.5889 | 0.7783 | 0.5485 | C | -5.3904 | 1.4045 | 0.5434 |
| C | -6.5079 | 1.9487 | 0.1844 | C | -6.3426 | 2.5058 | 0.0613 |
| H | -3.6446 | 3.2128 | -0.6271 | H | -3.4888 | 3.7098 | -0.9385 |
| H | -3.4022 | 0.6291 | 0.3103 | H | -3.1817 | 1.3105 | 0.3437 |
| H | -3.9409 | 1.9634 | 1.3378 | H | -3.7968 | 2.7515 | 1.1906 |
| H | -5.7076 | -0.0241 | -0.1818 | H | -5.4815 | 0.5245 | -0.1109 |
| H | -5.8095 | 0.3808 | 1.5393 | H | -5.6076 | 1.0928 | 1.5744 |
| H | -7.432 | 1.6185 | -0.2891 | H | -7.2595 | 2.0984 | -0.3863 |
| H | -6.7542 | 2.5532 | 1.0627 | H | -6.6219 | 3.1871 | 0.8823 |
| N | -2.6973 | 1.7848 | -2.5178 | N | -2.6006 | 1.9453 | -2.5897 |
| C | -2.2429 | 1.2704 | -3.797 | C | -2.1841 | 1.3245 | -3.828 |
| C | -1.0289 | 0.3687 | -3.5793 | C | -1.0408 | 0.3506 | -3.5511 |
| O | -0.2151 | 0.6161 | -2.6655 | O | -0.2439 | 0.5745 | -2.6128 |
| C | -1.8778 | 2.423 | -4.7564 | C | -1.7124 | 2.3832 | -4.8478 |
| C | -3.0967 | 3.3046 | -5.0786 | C | -2.8037 | 3.4032 | -5.2005 |
| C | -2.6671 | 4.509 | -5.9014 | C | -2.203 | 4.5342 | -6.0263 |
| O | -2.443 | 4.4157 | -7.1109 | O | -1.8868 | 4.3712 | -7.2011 |
| N | -2.5128 | 5.6637 | -5.2176 | N | -1.9921 | 5.6916 | -5.3548 |
| H | -2.0635 | 2.4133 | -2.0306 | H | -1.9129 | 2.5463 | -2.1277 |
| H | -3.0619 | 0.6864 | -4.2186 | H | -3.0498 | 0.7786 | -4.2285 |
| H | -1.0949 | 3.025 | -4.2897 | H | -0.8469 | 2.9065 | -4.4136 |
| H | -1.4675 | 2.0069 | -5.6796 | H | -1.3606 | 1.8803 | -5.7617 |
| H | -3.8182 | 2.7365 | -5.6696 | H | -3.5878 | 2.9203 | -5.8033 |
| H | -3.5909 | 3.6198 | -4.1576 | H | -3.2763 | 3.7898 | -4.2865 |
| H | -2.6828 | 5.731 | -4.2189 | H | -2.2839 | 5.8261 | -4.3865 |
| H | -2.1775 | 6.479 | -5.7094 | H | -1.5631 | 6.4665 | -5.8494 |
| N | -0.8857 | -0.6451 | -4.4259 | N | -0.9225 | -0.6768 | -4.379 |
| C | 0.2813 | -1.5149 | -4.5185 | C | 0.1978 | -1.5968 | -4.4639 |
| C | 0.5657 | -1.8351 | -6.0281 | C | 0.4001 | -1.9666 | -5.9874 |
| O | -0.3683 | -1.5962 | -6.8318 | O | -0.4975 | -1.5575 | -6.7622 |
| C | 0.0661 | -2.846 | -3.7882 | C | -0.0301 | -2.8961 | -3.6909 |
| S | -0.2285 | -2.7505 | -1.9703 | S | -0.2397 | -2.7743 | -1.8646 |
| O | 1.691 | -2.3284 | -6.2598 | O | 1.4087 | -2.6513 | -6.2336 |
| H | -1.5149 | -0.7359 | -5.2188 | H | -1.4874 | -0.7282 | -5.2353 |
| H | 1.1452 | -0.9934 | -4.1103 | H | 1.1131 | -1.1054 | -4.1036 |
| H | -0.8329 | -3.3397 | -4.1687 | H | -0.9681 | -3.3715 | -4.0248 |
| H | 0.9199 | -3.5009 | -3.9572 | H | 0.7997 | -3.584 | -3.9008 |
| H | -0.3416 | 0.8186 | 0.5357 | H | -0.35 | 0.8289 | 0.529 |
| H | 3.6776 | 5.6978 | 7.0969 | H | 2.8849 | 4.6541 | 7.4951 |

Table S6. Cartesian coordinates of Ptry9L optimized at B3LYP-D3/6-311+G(d,p) and B3LYP-D3/Def2-SVP

|  | B3LYP-D3/6-311+G(d,p) | | | B3LYP-D3/Def2-SVP | | | |
| --- | --- | --- | --- | --- | --- | --- | --- |
| Symbol | X | Y | Z | Symbol | X | Y | Z |
| N | 0 | 0 | 0 | N | 0 | 0 | 0 |
| C | 1.5012 | 0 | 0 | C | 1.4933 | 0 | 0 |
| C | 1.9534 | 1.4695 | 0 | C | 1.9393 | 1.4704 | 0 |
| O | 1.2476 | 2.3255 | -0.5358 | O | 1.2255 | 2.3145 | -0.5415 |
| C | 2.043 | -0.6723 | -1.2636 | C | 2.0425 | -0.6275 | -1.2848 |
| S | 1.5589 | -2.4333 | -1.5 | S | 1.592 | -2.3811 | -1.5921 |
| H | -0.3686 | -0.9129 | -0.2859 | H | -0.3811 | -0.885 | -0.3643 |
| H | -0.3406 | 0.7278 | -0.6411 | H | -0.321 | 0.7922 | -0.5857 |
| H | 1.8431 | -0.5199 | 0.8915 | H | 1.8592 | -0.5395 | 0.8813 |
| H | 1.6829 | -0.1565 | -2.1576 | H | 1.6699 | -0.0749 | -2.1629 |
| H | 3.1292 | -0.6039 | -1.255 | H | 3.1346 | -0.5224 | -1.2797 |
| N | 3.1529 | 1.6786 | 0.5462 | N | 3.1566 | 1.6903 | 0.5082 |
| C | 3.8184 | 2.9611 | 0.4628 | C | 3.847 | 2.9332 | 0.2536 |
| C | 4.304 | 3.2394 | -0.9694 | C | 4.2375 | 3.027 | -1.2299 |
| O | 4.5545 | 2.3423 | -1.7697 | O | 4.5149 | 2.0314 | -1.8966 |
| C | 5.0331 | 2.9827 | 1.4248 | C | 5.1325 | 3.0204 | 1.1127 |
| O | 5.9077 | 1.9025 | 1.1002 | O | 5.9662 | 1.916 | 0.8152 |
| C | 4.6094 | 2.8665 | 2.8849 | C | 4.8272 | 3.0446 | 2.6066 |
| H | 3.6318 | 0.8928 | 0.9922 | H | 3.6601 | 0.8968 | 0.9315 |
| H | 3.1192 | 3.7471 | 0.7611 | H | 3.1854 | 3.776 | 0.5077 |
| H | 5.5522 | 3.9337 | 1.2748 | H | 5.6398 | 3.9602 | 0.8341 |
| H | 6.7221 | 2.2124 | 0.6572 | H | 6.7687 | 2.1784 | 0.3105 |
| H | 5.4939 | 2.9204 | 3.522 | H | 5.7725 | 3.1132 | 3.1639 |
| H | 3.931 | 3.6791 | 3.155 | H | 4.1928 | 3.9052 | 2.8667 |
| H | 4.1082 | 1.9145 | 3.0703 | H | 4.3109 | 2.1225 | 2.9123 |
| N | 4.4984 | 4.5513 | -1.2354 | N | 4.3258 | 4.2899 | -1.7043 |
| C | 5.1487 | 5.0374 | -2.4533 | C | 4.8522 | 4.6167 | -3.0264 |
| C | 6.676 | 5.0761 | -2.2174 | C | 6.3878 | 4.7772 | -2.9394 |
| O | 7.2999 | 6.1257 | -2.0839 | O | 6.9567 | 5.8388 | -3.1563 |
| C | 4.5921 | 6.3964 | -2.8691 | C | 4.1746 | 5.8553 | -3.6029 |
| C | 3.1096 | 6.333 | -3.2474 | C | 2.6712 | 5.67 | -3.8156 |
| C | 2.5687 | 7.703 | -3.6721 | C | 2.0096 | 6.9264 | -4.387 |
| C | 1.0958 | 7.6151 | -4.045 | C | 0.5137 | 6.733 | -4.5851 |
| N | 0.5646 | 8.963 | -4.4803 | N | -0.1195 | 7.9629 | -5.1737 |
| H | 4.3068 | 5.2311 | -0.5121 | H | 4.1304 | 5.0647 | -1.0775 |
| H | 4.9419 | 4.2998 | -3.2323 | H | 4.6444 | 3.7437 | -3.6657 |
| H | 4.751 | 7.117 | -2.0602 | H | 4.3697 | 6.715 | -2.9391 |
| H | 5.1797 | 6.7548 | -3.7183 | H | 4.6672 | 6.0969 | -4.5573 |
| H | 2.9741 | 5.6168 | -4.0657 | H | 2.5002 | 4.8192 | -4.4981 |
| H | 2.5256 | 5.9569 | -2.4014 | H | 2.189 | 5.3998 | -2.8609 |
| H | 2.6991 | 8.4199 | -2.8542 | H | 2.184 | 7.7783 | -3.7072 |
| H | 3.1425 | 8.0776 | -4.5266 | H | 2.4786 | 7.1864 | -5.3518 |
| H | 0.9271 | 6.9365 | -4.881 | H | 0.2957 | 5.9063 | -5.2751 |
| H | 0.4804 | 7.2993 | -3.2032 | H | -0.0024 | 6.5366 | -3.6355 |
| H | -0.4247 | 8.9125 | -4.7343 | H | -1.1319 | 7.8476 | -5.311 |
| H | 0.6533 | 9.6605 | -3.7375 | H | 0.0108 | 8.7866 | -4.5707 |
| N | 7.2434 | 3.8513 | -2.1405 | N | 7.0228 | 3.6372 | -2.5638 |
| C | 8.6632 | 3.6498 | -1.9431 | C | 8.4564 | 3.5285 | -2.4559 |
| C | 9.1114 | 2.4686 | -2.8134 | C | 8.9364 | 2.3665 | -3.3425 |
| O | 8.3037 | 1.6421 | -3.2308 | O | 8.1493 | 1.5373 | -3.7882 |
| C | 9.0275 | 3.4099 | -0.4613 | C | 8.9139 | 3.3553 | -0.9926 |
| O | 8.3965 | 2.2582 | 0.0962 | O | 8.3425 | 2.2206 | -0.3644 |
| H | 6.678 | 3.0272 | -2.311 | H | 6.4891 | 2.77 | -2.5386 |
| H | 9.1827 | 4.5589 | -2.257 | H | 8.8926 | 4.4693 | -2.8265 |
| H | 10.1142 | 3.3361 | -0.3555 | H | 10.0157 | 3.3125 | -0.9439 |
| H | 8.6829 | 4.2672 | 0.1181 | H | 8.5855 | 4.2389 | -0.4252 |
| H | 8.7915 | 1.4261 | -0.2561 | H | 8.7394 | 1.3729 | -0.708 |
| N | 10.4421 | 2.3982 | -3.0272 | N | 10.2731 | 2.3196 | -3.5462 |
| C | 11.0978 | 1.2397 | -3.6273 | C | 10.9458 | 1.2002 | -4.1905 |
| C | 12.0969 | 0.7284 | -2.5749 | C | 11.9307 | 0.6364 | -3.15 |
| O | 13.0336 | 1.4586 | -2.2354 | O | 12.8809 | 1.3308 | -2.7922 |
| C | 11.8088 | 1.5712 | -4.9578 | C | 11.6874 | 1.603 | -5.4849 |
| C | 10.8034 | 2.1382 | -5.9754 | C | 10.7219 | 2.2733 | -6.4768 |
| C | 12.5231 | 0.3185 | -5.4861 | C | 12.3871 | 0.3769 | -6.0849 |
| C | 11.4531 | 2.6954 | -7.2461 | C | 11.3986 | 2.8671 | -7.7137 |
| H | 11.0483 | 3.1143 | -2.6503 | H | 10.8738 | 3.0348 | -3.1474 |
| H | 10.3071 | 0.5217 | -3.8305 | H | 10.1573 | 0.4872 | -4.4542 |
| H | 12.5599 | 2.3396 | -4.7395 | H | 12.4591 | 2.3374 | -5.1916 |
| H | 10.0851 | 1.3519 | -6.2392 | H | 9.9567 | 1.5366 | -6.783 |
| H | 10.2278 | 2.9359 | -5.4972 | H | 10.1795 | 3.0753 | -5.9503 |
| H | 13.0697 | 0.5373 | -6.4046 | H | 12.9648 | 0.6464 | -6.9806 |
| H | 13.2443 | -0.0728 | -4.7644 | H | 13.0889 | -0.0834 | -5.3724 |
| H | 11.7999 | -0.4739 | -5.708 | H | 11.6492 | -0.389 | -6.3788 |
| H | 10.7003 | 3.1467 | -7.898 | H | 10.6684 | 3.4011 | -8.3422 |
| H | 12.1891 | 3.4678 | -7.001 | H | 12.1834 | 3.5872 | -7.4268 |
| H | 11.9627 | 1.9189 | -7.822 | H | 11.8696 | 2.0949 | -8.3421 |
| N | 11.9043 | -0.4927 | -2.0425 | N | 11.7082 | -0.6006 | -2.6495 |
| C | 10.7711 | -1.3836 | -2.3054 | C | 10.5164 | -1.4168 | -2.868 |
| C | 9.4786 | -0.837 | -1.6797 | C | 9.2909 | -0.792 | -2.1862 |
| O | 9.535 | -0.055 | -0.7138 | O | 9.4546 | -0.0112 | -1.2309 |
| C | 11.1927 | -2.6951 | -1.6026 | C | 10.8689 | -2.747 | -2.1612 |
| C | 12.0189 | -2.2018 | -0.4064 | C | 11.7448 | -2.2873 | -0.9878 |
| C | 12.7816 | -0.9971 | -0.9665 | C | 12.5661 | -1.1376 | -1.5804 |
| H | 10.6459 | -1.5389 | -3.3752 | H | 10.3334 | -1.571 | -3.9386 |
| H | 10.3344 | -3.3025 | -1.3148 | H | 9.97 | -3.2948 | -1.8448 |
| H | 11.8118 | -3.2774 | -2.2888 | H | 11.4383 | -3.3833 | -2.8561 |
| H | 11.3554 | -1.8812 | 0.3992 | H | 11.1074 | -1.9107 | -0.174 |
| H | 12.6886 | -2.9684 | -0.0162 | H | 12.3763 | -3.0919 | -0.5861 |
| H | 12.9533 | -0.211 | -0.2309 | H | 12.8018 | -0.3449 | -0.8562 |
| H | 13.7479 | -1.2827 | -1.3928 | H | 13.5192 | -1.4875 | -2.0126 |
| N | 8.3246 | -1.2825 | -2.1815 | N | 8.0859 | -1.1795 | -2.6079 |
| C | 7.0519 | -0.9051 | -1.5603 | C | 6.8852 | -0.7755 | -1.8787 |
| C | 6.8985 | -1.6506 | -0.2247 | C | 6.8369 | -1.5517 | -0.5513 |
| O | 7.3084 | -2.8023 | -0.0856 | O | 7.2804 | -2.6938 | -0.4621 |
| C | 6.0024 | -1.3718 | -2.5877 | C | 5.7395 | -1.1834 | -2.8225 |
| C | 6.6689 | -2.5772 | -3.2654 | C | 6.3064 | -2.404 | -3.5566 |
| C | 8.1469 | -2.1789 | -3.3442 | C | 7.7833 | -2.0475 | -3.7577 |
| H | 7.0203 | 0.1736 | -1.411 | H | 6.8922 | 0.3063 | -1.696 |
| H | 5.0526 | -1.6241 | -2.1168 | H | 4.8127 | -1.4042 | -2.2772 |
| H | 5.8277 | -0.5662 | -3.304 | H | 5.5393 | -0.3517 | -3.5143 |
| H | 6.5596 | -3.4634 | -2.6384 | H | 6.226 | -3.2926 | -2.9134 |
| H | 6.2531 | -2.7934 | -4.2497 | H | 5.7973 | -2.6144 | -4.5074 |
| H | 8.8107 | -3.0407 | -3.2812 | H | 8.4274 | -2.9371 | -3.7712 |
| H | 8.3656 | -1.6287 | -4.2646 | H | 7.9419 | -1.4836 | -4.6924 |
| N | 6.259 | -0.9549 | 0.7347 | N | 6.2513 | -0.8806 | 0.459 |
| C | 5.9939 | -1.485 | 2.0678 | C | 6.0721 | -1.4309 | 1.7938 |
| C | 4.4735 | -1.6456 | 2.1628 | C | 4.5634 | -1.6402 | 1.9529 |
| O | 3.7382 | -0.6642 | 1.9732 | O | 3.7948 | -0.6821 | 1.7713 |
| C | 6.5355 | -0.5344 | 3.1459 | C | 6.6358 | -0.4724 | 2.8526 |
| C | 8.0496 | -0.3024 | 2.9864 | C | 8.1194 | -0.1524 | 2.6112 |
| C | 8.5025 | 0.8763 | 3.8306 | C | 8.5473 | 1.0626 | 3.4216 |
| O | 8.5669 | 0.8047 | 5.0609 | O | 8.6314 | 1.0248 | 4.6464 |
| N | 8.8064 | 2.0044 | 3.1511 | N | 8.7881 | 2.1823 | 2.6981 |
| H | 5.9923 | 0.0145 | 0.5824 | H | 5.9475 | 0.0892 | 0.3362 |
| H | 6.4863 | -2.4556 | 2.1309 | H | 6.5977 | -2.3951 | 1.819 |
| H | 6.0071 | 0.414 | 3.0577 | H | 6.0509 | 0.4559 | 2.809 |
| H | 6.3178 | -0.951 | 4.1322 | H | 6.4952 | -0.9087 | 3.8534 |
| H | 8.6023 | -1.185 | 3.3178 | H | 8.7456 | -1.0039 | 2.9213 |
| H | 8.2927 | -0.1333 | 1.936 | H | 8.2957 | 0.0152 | 1.5395 |
| H | 8.6954 | 2.0801 | 2.1455 | H | 8.675 | 2.213 | 1.6862 |
| H | 9.0523 | 2.8334 | 3.6721 | H | 9.0273 | 3.0367 | 3.19 |
| N | 4.0065 | -2.8766 | 2.3933 | N | 4.1386 | -2.8786 | 2.2112 |
| C | 2.6 | -3.2402 | 2.316 | C | 2.7511 | -3.2967 | 2.1817 |
| C | 2.147 | -4.0594 | 3.5687 | C | 2.4219 | -4.2255 | 3.4068 |
| O | 3.0507 | -4.6621 | 4.1952 | O | 3.4108 | -4.7793 | 3.9388 |
| C | 2.3366 | -4.1121 | 1.077 | C | 2.4573 | -4.1187 | 0.9174 |
| S | 3.0564 | -3.525 | -0.5208 | S | 3.1139 | -3.4885 | -0.6888 |
| O | 0.9119 | -4.063 | 3.7824 | O | 1.2075 | -4.3527 | 3.6596 |
| H | 4.6468 | -3.617 | 2.6476 | H | 4.7896 | -3.5991 | 2.5175 |
| H | 2.0173 | -2.324 | 2.2654 | H | 2.1092 | -2.4081 | 2.2169 |
| H | 2.8166 | -5.0867 | 1.2056 | H | 2.9689 | -5.0929 | 1.0004 |
| H | 1.2684 | -4.2765 | 0.9424 | H | 1.3802 | -4.3123 | 0.8235 |
| H | 1.071 | 9.3189 | -5.2948 | H | 0.2831 | 8.1978 | -6.0912 |
| H | -0.3748 | 0.2091 | 0.9284 | H | -0.3851 | 0.1411 | 0.9425 |

Table S7. Cartesian coordinates of Ptry6 optimized at B3LYP/6-311+G(d,p) and B3LYP/Def2-SVP

|  | B3LYP/6-311+G(d,p) | | | B3LYP/Def2-SVP | | |
| --- | --- | --- | --- | --- | --- | --- |
| Symbol | X | Y | Z | X | Y | Z |
| N | 7.7639 | -2.3585 | -1.7306 | 7.7639 | -2.3585 | -1.7306 |
| C | 7.1749 | -1.1803 | -1.073 | 7.1749 | -1.1803 | -1.073 |
| C | 5.6542 | -1.3565 | -0.9194 | 5.6542 | -1.3565 | -0.9194 |
| O | 4.8747 | -1.1287 | -1.8509 | 4.8747 | -1.1287 | -1.8509 |
| C | 7.5301 | 0.0551 | -1.8999 | 7.5301 | 0.0551 | -1.8999 |
| S | 7.0312 | 1.6559 | -1.1475 | 7.0312 | 1.6559 | -1.1475 |
| H | 7.6744 | -3.1542 | -1.1315 | 7.6744 | -3.1542 | -1.1315 |
| H | 7.2857 | -2.5337 | -2.5912 | 7.2857 | -2.5337 | -2.5912 |
| H | 7.6318 | -1.0903 | -0.0845 | 7.6318 | -1.0903 | -0.0845 |
| H | 8.6143 | 0.1062 | -1.9971 | 8.6143 | 0.1062 | -1.9971 |
| H | 7.0988 | -0.013 | -2.8985 | 7.0988 | -0.013 | -2.8985 |
| H | 5.7202 | 1.5802 | -1.4482 | 5.7202 | 1.5802 | -1.4482 |
| N | 5.2329 | -1.8621 | 0.2621 | 5.2329 | -1.8621 | 0.2621 |
| C | 3.8911 | -2.4014 | 0.4622 | 3.8911 | -2.4014 | 0.4622 |
| C | 2.8879 | -1.3271 | 0.9415 | 2.8879 | -1.3271 | 0.9415 |
| O | 2.332 | -1.4051 | 2.0438 | 2.332 | -1.4051 | 2.0438 |
| C | 3.9209 | -3.5914 | 1.4408 | 3.9209 | -3.5914 | 1.4408 |
| O | 4.4434 | -3.1753 | 2.709 | 4.4434 | -3.1753 | 2.709 |
| C | 4.7584 | -4.759 | 0.9436 | 4.7584 | -4.759 | 0.9436 |
| H | 5.8843 | -1.9634 | 1.029 | 5.8843 | -1.9634 | 1.029 |
| H | 3.5362 | -2.7552 | -0.5102 | 3.5362 | -2.7552 | -0.5102 |
| H | 2.8844 | -3.9213 | 1.5654 | 2.8844 | -3.9213 | 1.5654 |
| H | 3.8049 | -2.5359 | 3.0594 | 3.8049 | -2.5359 | 3.0594 |
| H | 4.6867 | -5.5862 | 1.6524 | 4.6867 | -5.5862 | 1.6524 |
| H | 4.3959 | -5.1052 | -0.0271 | 4.3959 | -5.1052 | -0.0271 |
| H | 5.8107 | -4.4843 | 0.8437 | 5.8107 | -4.4843 | 0.8437 |
| N | 2.6416 | -0.3366 | 0.0629 | 2.6416 | -0.3366 | 0.0629 |
| C | 1.6601 | 0.7239 | 0.3199 | 1.6601 | 0.7239 | 0.3199 |
| C | 0.2777 | 0.2615 | -0.1916 | 0.2777 | 0.2615 | -0.1916 |
| O | -0.2445 | 0.7116 | -1.2134 | -0.2445 | 0.7116 | -1.2134 |
| C | 2.0901 | 2.0633 | -0.28 | 2.0901 | 2.0633 | -0.28 |
| C | 3.2357 | 2.728 | 0.4917 | 3.2357 | 2.728 | 0.4917 |
| C | 3.6354 | 4.0875 | -0.0937 | 3.6354 | 4.0875 | -0.0937 |
| C | 4.7358 | 4.7893 | 0.7141 | 4.7358 | 4.7893 | 0.7141 |
| N | 5.1653 | 6.0999 | 0.2022 | 5.1653 | 6.0999 | 0.2022 |
| H | 3.1108 | -0.3754 | -0.8388 | 3.1108 | -0.3754 | -0.8388 |
| H | 1.5892 | 0.82 | 1.406 | 1.5892 | 0.82 | 1.406 |
| H | 2.362 | 1.9255 | -1.3319 | 2.362 | 1.9255 | -1.3319 |
| H | 1.2192 | 2.7241 | -0.2766 | 1.2192 | 2.7241 | -0.2766 |
| H | 2.9284 | 2.8624 | 1.5363 | 2.9284 | 2.8624 | 1.5363 |
| H | 4.1078 | 2.0651 | 0.5113 | 4.1078 | 2.0651 | 0.5113 |
| H | 3.977 | 3.96 | -1.1287 | 3.977 | 3.96 | -1.1287 |
| H | 2.7546 | 4.7409 | -0.1349 | 2.7546 | 4.7409 | -0.1349 |
| H | 4.3952 | 4.9296 | 1.7457 | 4.3952 | 4.9296 | 1.7457 |
| H | 5.6202 | 4.1454 | 0.7653 | 5.6202 | 4.1454 | 0.7653 |
| H | 5.5261 | 5.9938 | -0.7244 | 5.5261 | 5.9938 | -0.7244 |
| H | 4.3841 | 6.7239 | 0.1864 | 4.3841 | 6.7239 | 0.1864 |
| N | -0.3101 | -0.6873 | 0.5702 | -0.3101 | -0.6873 | 0.5702 |
| C | -1.53 | -1.384 | 0.1663 | -1.53 | -1.384 | 0.1663 |
| C | -2.7951 | -0.6051 | 0.5913 | -2.7951 | -0.6051 | 0.5913 |
| O | -3.5694 | -1.0468 | 1.4498 | -3.5694 | -1.0468 | 1.4498 |
| C | -1.5411 | -2.803 | 0.7371 | -1.5411 | -2.803 | 0.7371 |
| O | -1.4843 | -2.8137 | 2.1614 | -1.4843 | -2.8137 | 2.1614 |
| H | 0.1612 | -1.0085 | 1.4095 | 0.1612 | -1.0085 | 1.4095 |
| H | -1.5311 | -1.4484 | -0.926 | -1.5311 | -1.4484 | -0.926 |
| H | -2.4323 | -3.3239 | 0.3741 | -2.4323 | -3.3239 | 0.3741 |
| H | -0.6607 | -3.3391 | 0.3804 | -0.6607 | -3.3391 | 0.3804 |
| H | -2.298 | -2.3778 | 2.457 | -2.298 | -2.3778 | 2.457 |
| N | -2.9855 | 0.5673 | -0.0417 | -2.9855 | 0.5673 | -0.0417 |
| C | -4.1242 | 1.4456 | 0.248 | -4.1242 | 1.4456 | 0.248 |
| C | -5.291 | 1.0151 | -0.6747 | -5.291 | 1.0151 | -0.6747 |
| O | -5.5201 | 1.5512 | -1.7538 | -5.5201 | 1.5512 | -1.7538 |
| C | -3.7695 | 2.937 | 0.1003 | -3.7695 | 2.937 | 0.1003 |
| C | -2.5002 | 3.3304 | 0.8902 | -2.5002 | 3.3304 | 0.8902 |
| C | -4.9737 | 3.81 | 0.4909 | -4.9737 | 3.81 | 0.4909 |
| C | -2.5558 | 3.1233 | 2.4091 | -2.5558 | 3.1233 | 2.4091 |
| H | -2.3067 | 0.8368 | -0.7492 | -2.3067 | 0.8368 | -0.7492 |
| H | -4.4164 | 1.2416 | 1.2798 | -4.4164 | 1.2416 | 1.2798 |
| H | -3.5593 | 3.1159 | -0.96 | -3.5593 | 3.1159 | -0.96 |
| H | -1.6401 | 2.7879 | 0.4879 | -1.6401 | 2.7879 | 0.4879 |
| H | -2.3089 | 4.3884 | 0.6808 | -2.3089 | 4.3884 | 0.6808 |
| H | -4.713 | 4.8679 | 0.4029 | -4.713 | 4.8679 | 0.4029 |
| H | -5.8303 | 3.6233 | -0.1585 | -5.8303 | 3.6233 | -0.1585 |
| H | -5.2862 | 3.6287 | 1.5232 | -5.2862 | 3.6287 | 1.5232 |
| H | -1.6224 | 3.4595 | 2.8686 | -1.6224 | 3.4595 | 2.8686 |
| H | -3.3691 | 3.6891 | 2.8706 | -3.3691 | 3.6891 | 2.8706 |
| H | -2.6871 | 2.0709 | 2.676 | -2.6871 | 2.0709 | 2.676 |
| N | -6.0041 | -0.0343 | -0.1901 | -6.0041 | -0.0343 | -0.1901 |
| C | -7.0861 | -0.6579 | -0.919 | -7.0861 | -0.6579 | -0.919 |
| C | -8.4584 | -0.4705 | -0.2599 | -8.4584 | -0.4705 | -0.2599 |
| O | -9.4937 | -0.7437 | -0.8224 | -9.4937 | -0.7437 | -0.8224 |
| C | -6.86 | -2.1689 | -1.1483 | -6.86 | -2.1689 | -1.1483 |
| S | -5.3968 | -2.5642 | -2.1837 | -5.3968 | -2.5642 | -2.1837 |
| O | -8.3978 | -0.0047 | 0.9949 | -8.3978 | -0.0047 | 0.9949 |
| H | -5.6819 | -0.4625 | 0.6698 | -5.6819 | -0.4625 | 0.6698 |
| H | -7.1535 | -0.1554 | -1.885 | -7.1535 | -0.1554 | -1.885 |
| H | -6.6815 | -2.6737 | -0.1969 | -6.6815 | -2.6737 | -0.1969 |
| H | -7.7475 | -2.6064 | -1.6012 | -7.7475 | -2.6064 | -1.6012 |
| H | -5.9076 | -2.1415 | -3.3581 | -5.9076 | -2.1415 | -3.3581 |
| H | 5.8778 | 6.4727 | 0.7966 | 5.8778 | 6.4727 | 0.7966 |
| H | 8.7317 | -2.1879 | -1.9157 | 8.7317 | -2.1879 | -1.9157 |

Table S8. Cartesian coordinates of Ptry9D optimized at B3LYP/6-311+G(d,p) and B3LYP/Def2-SVP

|  | B3LYP/6-311+G(d,p) | | | B3LYP/Def2-SVP | | |
| --- | --- | --- | --- | --- | --- | --- |
| Symbol | X | Y | Z | X | Y | Z |
| N | -3.4501 | 1.7303 | -2.117 | -3.4742 | 1.6908 | -2.1057 |
| C | -4.1315 | 2.8484 | -1.4588 | -4.1543 | 2.8104 | -1.4488 |
| C | -3.2717 | 3.4039 | -0.2981 | -3.2912 | 3.3701 | -0.2926 |
| O | -3.7218 | 4.2735 | 0.4987 | -3.7392 | 4.2418 | 0.503 |
| C | -5.5214 | 2.5141 | -0.882 | -5.5421 | 2.477 | -0.8665 |
| S | -6.7911 | 1.8981 | -2.1662 | -6.8155 | 1.8562 | -2.1448 |
| H | -3.6645 | 0.8799 | -1.6365 | -3.6865 | 0.8417 | -1.6219 |
| H | -3.7587 | 1.6639 | -3.0659 | -3.7858 | 1.6213 | -3.0534 |
| H | -4.233 | 3.6593 | -2.1903 | -4.2586 | 3.619 | -2.1825 |
| H | -5.4378 | 1.7484 | -0.1125 | -5.4555 | 1.7137 | -0.0949 |
| H | -5.9976 | 3.4067 | -0.4684 | -6.0175 | 3.3706 | -0.4542 |
| N | -2.0322 | 2.8737 | -0.2332 | -2.0511 | 2.8409 | -0.2299 |
| C | -1.0303 | 3.2171 | 0.7525 | -1.0463 | 3.188 | 0.7515 |
| C | 0.318 | 3.3981 | 0.0343 | 0.2996 | 3.3676 | 0.0285 |
| O | 0.4782 | 3.0856 | -1.1713 | 0.4562 | 3.0515 | -1.1767 |
| C | -0.8833 | 2.1282 | 1.8576 | -0.895 | 2.1026 | 1.8595 |
| O | -0.4066 | 0.9163 | 1.1826 | -0.4197 | 0.8889 | 1.1867 |
| C | -2.1713 | 1.8531 | 2.6251 | -2.1804 | 1.8291 | 2.6319 |
| H | -1.7935 | 2.2325 | -0.9855 | -1.8143 | 2.1975 | -0.9811 |
| H | -1.3357 | 4.1537 | 1.2339 | -1.3507 | 4.1259 | 1.2309 |
| H | -0.1089 | 2.4641 | 2.5593 | -0.1187 | 2.4413 | 2.5577 |
| H | 0.2417 | 0.3628 | 1.7145 | 0.2307 | 0.3376 | 1.7182 |
| H | -1.9785 | 1.1107 | 3.4073 | -1.9846 | 1.0892 | 3.4158 |
| H | -2.5437 | 2.7675 | 3.0998 | -2.5519 | 2.7447 | 3.105 |
| H | -2.9389 | 1.4601 | 1.9539 | -2.9499 | 1.4335 | 1.9644 |
| N | 1.3381 | 3.856 | 0.8172 | 1.3219 | 3.8287 | 0.8067 |
| C | 2.7234 | 3.9853 | 0.3527 | 2.7056 | 3.9574 | 0.3374 |
| C | 3.6076 | 2.8175 | 0.8367 | 3.5922 | 2.7917 | 0.8222 |
| O | 4.8634 | 2.897 | 0.7968 | 4.8478 | 2.8719 | 0.778 |
| C | 3.332 | 5.3441 | 0.7502 | 3.3146 | 5.3178 | 0.7287 |
| C | 2.6696 | 6.5401 | 0.0474 | 2.6492 | 6.5112 | 0.0243 |
| C | 3.2928 | 7.884 | 0.4639 | 3.2727 | 7.8568 | 0.4347 |
| C | 2.6464 | 9.0859 | -0.2386 | 2.6234 | 9.0561 | -0.2695 |
| N | 3.2724 | 10.3484 | 0.193 | 3.2499 | 10.3203 | 0.1563 |
| H | 1.1375 | 4.1732 | 1.7573 | 1.1241 | 4.1486 | 1.7464 |
| H | 2.6861 | 3.9104 | -0.7415 | 2.6649 | 3.8791 | -0.7564 |
| H | 3.2623 | 5.4649 | 1.8415 | 3.2483 | 5.442 | 1.8199 |
| H | 4.3993 | 5.3072 | 0.5083 | 4.3811 | 5.2809 | 0.4836 |
| H | 2.7601 | 6.4147 | -1.042 | 2.7363 | 6.3825 | -1.0649 |
| H | 1.5937 | 6.5535 | 0.2719 | 1.574 | 6.5246 | 0.2522 |
| H | 3.2013 | 8.0206 | 1.55 | 3.1847 | 7.9967 | 1.5207 |
| H | 4.3704 | 7.8717 | 0.2378 | 4.3496 | 7.8445 | 0.2053 |
| H | 2.692 | 8.9267 | -1.3326 | 2.6656 | 8.8935 | -1.363 |
| H | 1.5823 | 9.1323 | 0.0257 | 1.56 | 9.1026 | -0.0019 |
| H | 2.767 | 11.1201 | -0.1929 | 2.7427 | 11.0905 | -0.2305 |
| H | 4.2196 | 10.3771 | -0.1263 | 4.196 | 10.3487 | -0.1662 |
| N | 2.9619 | 1.706 | 1.2739 | 2.9486 | 1.6811 | 1.2648 |
| C | 3.6704 | 0.4898 | 1.6307 | 3.659 | 0.4666 | 1.6232 |
| C | 4.0344 | -0.3123 | 0.3645 | 4.0195 | -0.3392 | 0.3583 |
| O | 3.4928 | -0.0777 | -0.7446 | 3.4742 | -0.1084 | -0.7498 |
| C | 2.8034 | -0.3441 | 2.6005 | 2.7956 | -0.365 | 2.5983 |
| O | 1.5084 | -0.6741 | 2.0276 | 1.4991 | -0.6976 | 2.0305 |
| H | 1.9591 | 1.6352 | 1.1624 | 1.9454 | 1.6093 | 1.1568 |
| H | 4.5955 | 0.7689 | 2.1471 | 4.5856 | 0.7478 | 2.1357 |
| H | 3.3289 | -1.2598 | 2.8883 | 3.3227 | -1.2794 | 2.8873 |
| H | 2.6059 | 0.2449 | 3.4976 | 2.6006 | 0.2267 | 3.4942 |
| H | 1.5604 | -1.4413 | 1.3835 | 1.5495 | -1.4668 | 1.3886 |
| N | 4.9441 | -1.3055 | 0.5491 | 4.9305 | -1.3313 | 0.5431 |
| C | 5.2667 | -2.299 | -0.4894 | 5.2504 | -2.3278 | -0.4933 |
| C | 4.9051 | -3.6668 | 0.1103 | 4.8916 | -3.694 | 0.1117 |
| O | 5.5933 | -4.1115 | 1.0742 | 5.5831 | -4.1352 | 1.0748 |
| C | 6.7617 | -2.247 | -0.9133 | 6.744 | -2.2761 | -0.9222 |
| C | 7.1322 | -0.8417 | -1.4429 | 7.1119 | -0.8722 | -1.4573 |
| C | 7.0597 | -3.3506 | -1.9485 | 7.0395 | -3.3827 | -1.9549 |
| C | 8.6434 | -0.6284 | -1.634 | 8.6223 | -0.6585 | -1.6539 |
| H | 5.3752 | -1.4474 | 1.4544 | 5.3645 | -1.4701 | 1.4474 |
| H | 4.645 | -2.0465 | -1.3487 | 4.6258 | -2.0783 | -1.3515 |
| H | 7.3552 | -2.4489 | -0.0097 | 7.3405 | -2.4748 | -0.0199 |
| H | 6.6106 | -0.6704 | -2.3964 | 6.5871 | -0.7042 | -2.4096 |
| H | 6.7561 | -0.087 | -0.7411 | 6.7376 | -0.1156 | -0.7566 |
| H | 8.1157 | -3.3404 | -2.2346 | 8.0945 | -3.3727 | -2.2444 |
| H | 6.8389 | -4.3475 | -1.5521 | 6.8206 | -4.3785 | -1.5548 |
| H | 6.4649 | -3.203 | -2.8594 | 6.4417 | -3.2383 | -2.8643 |
| H | 8.8508 | 0.3998 | -1.9521 | 8.828 | 0.3688 | -1.9759 |
| H | 9.1855 | -0.8039 | -0.6961 | 9.1675 | -0.8308 | -0.7172 |
| H | 9.0613 | -1.2985 | -2.3934 | 9.0383 | -1.3307 | -2.4125 |
| N | 3.8422 | -4.3587 | -0.3717 | 3.8277 | -4.3881 | -0.3647 |
| C | 2.8946 | -3.9353 | -1.4198 | 2.8764 | -3.9685 | -1.4112 |
| C | 1.9214 | -2.8627 | -0.9075 | 1.9042 | -2.8949 | -0.8991 |
| O | 1.7306 | -2.7248 | 0.3458 | 1.7173 | -2.7533 | 0.3544 |
| C | 2.1224 | -5.252 | -1.7344 | 2.1041 | -5.2867 | -1.7192 |
| C | 2.0976 | -5.9871 | -0.3781 | 2.0841 | -6.0176 | -0.3606 |
| C | 3.4574 | -5.6533 | 0.256 | 3.4457 | -5.6809 | 0.2681 |
| H | 3.4281 | -3.5764 | -2.3018 | 3.4069 | -3.612 | -2.296 |
| H | 1.1214 | -5.0579 | -2.1273 | 1.1017 | -5.0944 | -2.1095 |
| H | 2.6839 | -5.8243 | -2.4799 | 2.6636 | -5.8609 | -2.4648 |
| H | 1.2845 | -5.6005 | 0.2444 | 1.2728 | -5.6296 | 0.2632 |
| H | 1.9524 | -7.0638 | -0.4952 | 1.9393 | -7.0948 | -0.4739 |
| H | 3.4128 | -5.5387 | 1.3416 | 3.4045 | -5.563 | 1.3536 |
| H | 4.2205 | -6.4059 | 0.0269 | 4.2086 | -6.4337 | 0.0389 |
| N | 1.245 | -2.1489 | -1.8216 | 1.2244 | -2.1844 | -1.8132 |
| C | 0.1825 | -1.2034 | -1.4134 | 0.1626 | -1.2384 | -1.4046 |
| C | -1.0424 | -1.9985 | -0.9338 | -1.0603 | -2.0328 | -0.9186 |
| O | -1.3301 | -3.1219 | -1.429 | -1.3488 | -3.1579 | -1.4094 |
| C | -0.0973 | -0.4039 | -2.7113 | -0.1219 | -0.4431 | -2.7041 |
| C | 0.2224 | -1.4059 | -3.839 | 0.1949 | -1.4484 | -3.8297 |
| C | 1.4276 | -2.1976 | -3.3007 | 1.4023 | -2.2376 | -3.2928 |
| H | 0.5467 | -0.5498 | -0.6205 | 0.5289 | -0.5821 | -0.6149 |
| H | -1.1208 | -0.0225 | -2.749 | -1.1458 | -0.0625 | -2.7397 |
| H | 0.5806 | 0.4556 | -2.7451 | 0.5553 | 0.4168 | -2.7427 |
| H | -0.6247 | -2.08 | -3.9975 | -0.6523 | -2.1235 | -3.9834 |
| H | 0.4525 | -0.9133 | -4.7869 | 0.4216 | -0.9586 | -4.7798 |
| H | 1.4405 | -3.2277 | -3.6623 | 1.4147 | -3.2688 | -3.6512 |
| H | 2.3758 | -1.7158 | -3.566 | 2.3494 | -1.7559 | -3.5626 |
| N | -1.7782 | -1.3969 | 0.0366 | -1.7934 | -1.4287 | 0.0522 |
| C | -2.9399 | -2.053 | 0.6379 | -2.9527 | -2.0837 | 0.6592 |
| C | -4.2127 | -1.2489 | 0.3636 | -4.2269 | -1.2813 | 0.3865 |
| O | -4.2048 | 0.0105 | 0.4218 | -4.2197 | -0.0217 | 0.4408 |
| C | -2.7672 | -2.1807 | 2.1752 | -2.7751 | -2.2065 | 2.1963 |
| C | -1.5441 | -3.0155 | 2.5784 | -1.5501 | -3.0393 | 2.5983 |
| C | -1.1568 | -2.7862 | 4.0338 | -1.1583 | -2.8052 | 4.0517 |
| O | -1.2955 | -1.6681 | 4.5983 | -1.2961 | -1.6854 | 4.6132 |
| N | -0.6202 | -3.853 | 4.6802 | -0.619 | -3.8697 | 4.6997 |
| H | -1.4714 | -0.4947 | 0.4206 | -1.486 | -0.525 | 0.4325 |
| H | -3.0113 | -3.043 | 0.1791 | -3.0249 | -3.0752 | 0.2038 |
| H | -2.6684 | -1.178 | 2.6024 | -2.6755 | -1.2024 | 2.6202 |
| H | -3.6788 | -2.6228 | 2.5956 | -3.6851 | -2.6479 | 2.621 |
| H | -1.718 | -4.0809 | 2.3903 | -1.7239 | -4.1053 | 2.414 |
| H | -0.6792 | -2.7203 | 1.968 | -0.6874 | -2.7454 | 1.9842 |
| H | -0.5166 | -4.7543 | 4.2404 | -0.5162 | -4.7723 | 4.2623 |
| H | -0.3023 | -3.7495 | 5.6332 | -0.2982 | -3.763 | 5.6514 |
| N | -5.345 | -1.9612 | 0.1267 | -5.3595 | -1.995 | 0.1554 |
| C | -6.6617 | -1.3373 | 0.0605 | -6.6769 | -1.3722 | 0.0915 |
| C | -7.6783 | -2.0646 | 0.9358 | -7.6901 | -2.0974 | 0.9722 |
| O | -7.2454 | -3.2933 | 1.3471 | -7.2551 | -3.3246 | 1.386 |
| C | -7.2487 | -1.2518 | -1.3684 | -7.2685 | -1.2915 | -1.3358 |
| S | -6.211 | -0.2433 | -2.5948 | -6.2353 | -0.2861 | -2.5686 |
| O | -8.7939 | -1.6091 | 1.2041 | -8.8052 | -1.6419 | 1.2426 |
| H | -5.3089 | -2.972 | 0.1073 | -5.3228 | -3.0059 | 0.139 |
| H | -6.5619 | -0.3256 | 0.4634 | -6.5764 | -0.3592 | 0.491 |
| H | -7.2933 | -2.2428 | -1.8306 | -7.3138 | -2.284 | -1.7948 |
| H | -8.2522 | -0.8252 | -1.3381 | -8.2721 | -0.8654 | -1.3036 |
| H | 3.2554 | 10.4046 | 1.1913 | 3.236 | 10.3796 | 1.1544 |
| H | -2.4622 | 1.8842 | -2.0978 | -2.4864 | 1.8453 | -2.0901 |

Table S9. Cartesian coordinates of Ptry9L optimized at B3LYP/6-311+G(d,p) and B3LYP/Def2-SVP

|  | B3LYP/6-311+G(d,p) | | | B3LYP/Def2-SVP | | |
| --- | --- | --- | --- | --- | --- | --- |
| Symbol | X | Y | Z | X | Y | Z |
| N | -6.031 | 2.4932 | -0.7393 | -6.031 | 2.4932 | -0.7393 |
| C | -4.8576 | 1.628 | -0.6733 | -4.8576 | 1.628 | -0.6733 |
| C | -3.752 | 2.3866 | 0.0757 | -3.752 | 2.3866 | 0.0757 |
| O | -3.615 | 3.6345 | -0.057 | -3.615 | 3.6345 | -0.057 |
| C | -4.2808 | 1.2042 | -2.0444 | -4.2808 | 1.2042 | -2.0444 |
| S | -5.5702 | 0.2903 | -3.1307 | -5.5702 | 0.2903 | -3.1307 |
| H | -6.7387 | 2.0535 | -1.2924 | -6.7387 | 2.0535 | -1.2924 |
| H | -5.778 | 3.3668 | -1.1549 | -5.778 | 3.3668 | -1.1549 |
| H | -5.1072 | 0.7257 | -0.1114 | -5.1072 | 0.7257 | -0.1114 |
| H | -3.9995 | 2.0817 | -2.6335 | -3.9995 | 2.0817 | -2.6335 |
| H | -3.4203 | 0.5383 | -1.9493 | -3.4203 | 0.5383 | -1.9493 |
| N | -2.927 | 1.6428 | 0.854 | -2.927 | 1.6428 | 0.854 |
| C | -1.8706 | 2.287 | 1.6214 | -1.8706 | 2.287 | 1.6214 |
| C | -0.7152 | 2.7541 | 0.7248 | -0.7152 | 2.7541 | 0.7248 |
| O | -0.2993 | 2.0805 | -0.2588 | -0.2993 | 2.0805 | -0.2588 |
| C | -1.3506 | 1.3762 | 2.7692 | -1.3506 | 1.3762 | 2.7692 |
| O | -0.722 | 0.1568 | 2.2574 | -0.722 | 0.1568 | 2.2574 |
| C | -2.4682 | 0.9768 | 3.7312 | -2.4682 | 0.9768 | 3.7312 |
| H | -3.064 | 0.632 | 0.9144 | -3.064 | 0.632 | 0.9144 |
| H | -2.3014 | 3.1764 | 2.0979 | -2.3014 | 3.1764 | 2.0979 |
| H | -0.5919 | 1.9542 | 3.3118 | -0.5919 | 1.9542 | 3.3118 |
| H | 0.2739 | 0.2548 | 2.0938 | 0.2739 | 0.2548 | 2.0938 |
| H | -2.0521 | 0.4204 | 4.5753 | -2.0521 | 0.4204 | 4.5753 |
| H | -2.9765 | 1.8653 | 4.1197 | -2.9765 | 1.8653 | 4.1197 |
| H | -3.2074 | 0.3443 | 3.2316 | -3.2074 | 0.3443 | 3.2316 |
| N | -0.1311 | 3.917 | 1.1109 | -0.1311 | 3.917 | 1.1109 |
| C | 1.0357 | 4.5123 | 0.4272 | 1.0357 | 4.5123 | 0.4272 |
| C | 2.3192 | 3.8609 | 0.9867 | 2.3192 | 3.8609 | 0.9867 |
| O | 3.1285 | 4.4711 | 1.7319 | 3.1285 | 4.4711 | 1.7319 |
| C | 1.0307 | 6.0375 | 0.578 | 1.0307 | 6.0375 | 0.578 |
| C | -0.1485 | 6.7166 | -0.1415 | -0.1485 | 6.7166 | -0.1415 |
| C | -0.1419 | 8.2451 | 0.0337 | -0.1419 | 8.2451 | 0.0337 |
| C | -1.3152 | 8.9326 | -0.6779 | -1.3152 | 8.9326 | -0.6779 |
| N | -1.2647 | 10.3928 | -0.4859 | -1.2647 | 10.3928 | -0.4859 |
| H | -0.5032 | 4.4216 | 1.9052 | -0.5032 | 4.4216 | 1.9052 |
| H | 0.9492 | 4.2426 | -0.6319 | 0.9492 | 4.2426 | -0.6319 |
| H | 1.0269 | 6.2959 | 1.6459 | 1.0269 | 6.2959 | 1.6459 |
| H | 1.9773 | 6.419 | 0.1778 | 1.9773 | 6.419 | 0.1778 |
| H | -0.106 | 6.4718 | -1.2133 | -0.106 | 6.4718 | -1.2133 |
| H | -1.0993 | 6.3097 | 0.2307 | -1.0993 | 6.3097 | 0.2307 |
| H | -0.18 | 8.4886 | 1.1069 | -0.18 | 8.4886 | 1.1069 |
| H | 0.7975 | 8.6652 | -0.3506 | 0.7975 | 8.6652 | -0.3506 |
| H | -1.2538 | 8.7342 | -1.7555 | -1.2538 | 8.7342 | -1.7555 |
| H | -2.2609 | 8.4774 | -0.3278 | -2.2609 | 8.4774 | -0.3278 |
| H | -1.9731 | 10.8269 | -1.0424 | -1.9731 | 10.8269 | -1.0424 |
| H | -1.4199 | 10.6078 | 0.4783 | -1.4199 | 10.6078 | 0.4783 |
| N | 2.4649 | 2.5613 | 0.6216 | 2.4649 | 2.5613 | 0.6216 |
| C | 3.5121 | 1.695 | 1.1336 | 3.5121 | 1.695 | 1.1336 |
| C | 4.0527 | 0.8353 | -0.0142 | 4.0527 | 0.8353 | -0.0142 |
| O | 3.4143 | 0.7053 | -1.0917 | 3.4143 | 0.7053 | -1.0917 |
| C | 2.9715 | 0.8049 | 2.2836 | 2.9715 | 0.8049 | 2.2836 |
| O | 1.8379 | 0.0073 | 1.8577 | 1.8379 | 0.0073 | 1.8577 |
| H | 1.7665 | 2.1388 | 0.0141 | 1.7665 | 2.1388 | 0.0141 |
| H | 4.3067 | 2.3285 | 1.5422 | 4.3067 | 2.3285 | 1.5422 |
| H | 3.7625 | 0.1574 | 2.6774 | 3.7625 | 0.1574 | 2.6774 |
| H | 2.6156 | 1.4522 | 3.0871 | 2.6156 | 1.4522 | 3.0871 |
| H | 2.0918 | -0.832 | 1.3703 | 2.0918 | -0.832 | 1.3703 |
| N | 5.2211 | 0.1895 | 0.2365 | 5.2211 | 0.1895 | 0.2365 |
| C | 5.793 | -0.818 | -0.6736 | 5.793 | -0.818 | -0.6736 |
| C | 5.833 | -2.138 | 0.1138 | 5.833 | -2.138 | 0.1138 |
| O | 6.6467 | -2.2526 | 1.075 | 6.6467 | -2.2526 | 1.075 |
| C | 7.2111 | -0.424 | -1.1748 | 7.2111 | -0.424 | -1.1748 |
| C | 7.1779 | 0.942 | -1.8995 | 7.1779 | 0.942 | -1.8995 |
| C | 7.7836 | -1.5393 | -2.0731 | 7.7836 | -1.5393 | -2.0731 |
| C | 8.571 | 1.5177 | -2.2054 | 8.571 | 1.5177 | -2.2054 |
| H | 5.7073 | 0.3346 | 1.1127 | 5.7073 | 0.3346 | 1.1127 |
| H | 5.1165 | -0.8674 | -1.5268 | 5.1165 | -0.8674 | -1.5268 |
| H | 7.8498 | -0.3334 | -0.284 | 7.8498 | -0.3334 | -0.284 |
| H | 6.607 | 0.8372 | -2.8343 | 6.607 | 0.8372 | -2.8343 |
| H | 6.63 | 1.6604 | -1.2772 | 6.63 | 1.6604 | -1.2772 |
| H | 8.7918 | -1.2851 | -2.4132 | 8.7918 | -1.2851 | -2.4132 |
| H | 7.8502 | -2.4935 | -1.5399 | 7.8502 | -2.4935 | -1.5399 |
| H | 7.1568 | -1.6848 | -2.9626 | 7.1568 | -1.6848 | -2.9626 |
| H | 8.4862 | 2.5119 | -2.6589 | 8.4862 | 2.5119 | -2.6589 |
| H | 9.1635 | 1.6185 | -1.2872 | 9.1635 | 1.6185 | -1.2872 |
| H | 9.1345 | 0.8858 | -2.9006 | 9.1345 | 0.8858 | -2.9006 |
| N | 4.9865 | -3.1465 | -0.214 | 4.9865 | -3.1465 | -0.214 |
| C | 3.9169 | -3.1279 | -1.2288 | 3.9169 | -3.1279 | -1.2288 |
| C | 2.6887 | -2.347 | -0.7362 | 2.6887 | -2.347 | -0.7362 |
| O | 2.5085 | -2.1744 | 0.5149 | 2.5085 | -2.1744 | 0.5149 |
| C | 3.5709 | -4.6382 | -1.3836 | 3.5709 | -4.6382 | -1.3836 |
| C | 3.7994 | -5.2054 | 0.0336 | 3.7994 | -5.2054 | 0.0336 |
| C | 5.0034 | -4.4122 | 0.5705 | 5.0034 | -4.4122 | 0.5705 |
| H | 4.2826 | -2.7141 | -2.1696 | 4.2826 | -2.7141 | -2.1696 |
| H | 2.5514 | -4.7941 | -1.7453 | 2.5514 | -4.7941 | -1.7453 |
| H | 4.2645 | -5.0878 | -2.1015 | 4.2645 | -5.0878 | -2.1015 |
| H | 2.919 | -5.0256 | 0.6582 | 2.919 | -5.0256 | 0.6582 |
| H | 3.991 | -6.2812 | 0.0202 | 3.991 | -6.2812 | 0.0202 |
| H | 4.9307 | -4.1883 | 1.6375 | 4.9307 | -4.1883 | 1.6375 |
| H | 5.9548 | -4.9277 | 0.3977 | 5.9548 | -4.9277 | 0.3977 |
| N | 1.8036 | -1.9305 | -1.6554 | 1.8036 | -1.9305 | -1.6554 |
| C | 0.5374 | -1.2681 | -1.2603 | 0.5374 | -1.2681 | -1.2603 |
| C | -0.4583 | -2.3252 | -0.7683 | -0.4583 | -2.3252 | -0.7683 |
| O | -0.5684 | -3.4375 | -1.3513 | -0.5684 | -3.4375 | -1.3513 |
| C | 0.0636 | -0.6048 | -2.5784 | 0.0636 | -0.6048 | -2.5784 |
| C | 0.6196 | -1.5256 | -3.6836 | 0.6196 | -1.5256 | -3.6836 |
| C | 1.9817 | -1.9852 | -3.1351 | 1.9817 | -1.9852 | -3.1351 |
| H | 0.7283 | -0.52 | -0.4906 | 0.7283 | -0.52 | -0.4906 |
| H | -1.0234 | -0.5008 | -2.6192 | -1.0234 | -0.5008 | -2.6192 |
| H | 0.5019 | 0.3959 | -2.6403 | 0.5019 | 0.3959 | -2.6403 |
| H | -0.0373 | -2.3888 | -3.8211 | -0.0373 | -2.3888 | -3.8211 |
| H | 0.7231 | -1.0128 | -4.6431 | 0.7231 | -1.0128 | -4.6431 |
| H | 2.241 | -2.9943 | -3.4621 | 2.241 | -2.9943 | -3.4621 |
| H | 2.7835 | -1.2967 | -3.4256 | 2.7835 | -1.2967 | -3.4256 |
| N | -1.2228 | -1.9671 | 0.2961 | -1.2228 | -1.9671 | 0.2961 |
| C | -2.2609 | -2.8534 | 0.8314 | -2.2609 | -2.8534 | 0.8314 |
| C | -3.6283 | -2.2558 | 0.4922 | -3.6283 | -2.2558 | 0.4922 |
| O | -3.8917 | -1.0506 | 0.7672 | -3.8917 | -1.0506 | 0.7672 |
| C | -2.1164 | -3.0037 | 2.3644 | -2.1164 | -3.0037 | 2.3644 |
| C | -0.7448 | -3.5923 | 2.7707 | -0.7448 | -3.5923 | 2.7707 |
| C | -0.335 | -3.2359 | 4.1937 | -0.335 | -3.2359 | 4.1937 |
| O | -0.1461 | -4.1133 | 5.0784 | -0.1461 | -4.1133 | 5.0784 |
| N | -0.1689 | -1.9084 | 4.4341 | -0.1689 | -1.9084 | 4.4341 |
| H | -1.0776 | -1.083 | 0.7927 | -1.0776 | -1.083 | 0.7927 |
| H | -2.1357 | -3.8202 | 0.3375 | -2.1357 | -3.8202 | 0.3375 |
| H | -2.2553 | -2.0151 | 2.8106 | -2.2553 | -2.0151 | 2.8106 |
| H | -2.9243 | -3.6439 | 2.7373 | -2.9243 | -3.6439 | 2.7373 |
| H | -0.7536 | -4.6819 | 2.6914 | -0.7536 | -4.6819 | 2.6914 |
| H | 0.0287 | -3.2114 | 2.091 | 0.0287 | -3.2114 | 2.091 |
| H | -0.323 | -1.1998 | 3.718 | -0.323 | -1.1998 | 3.718 |
| H | 0.1104 | -1.6094 | 5.3575 | 0.1104 | -1.6094 | 5.3575 |
| N | -4.5251 | -3.0759 | -0.1047 | -4.5251 | -3.0759 | -0.1047 |
| C | -5.8559 | -2.6316 | -0.5056 | -5.8559 | -2.6316 | -0.5056 |
| C | -6.9487 | -3.4215 | 0.2078 | -6.9487 | -3.4215 | 0.2078 |
| O | -6.6071 | -4.7385 | 0.346 | -6.6071 | -4.7385 | 0.346 |
| C | -6.0866 | -2.7598 | -2.0264 | -6.0866 | -2.7598 | -2.0264 |
| S | -4.826 | -1.8382 | -3.106 | -4.826 | -1.8382 | -3.106 |
| O | -8.0321 | -2.9519 | 0.5639 | -8.0321 | -2.9519 | 0.5639 |
| H | -4.2829 | -4.0438 | -0.2731 | -4.2829 | -4.0438 | -0.2731 |
| H | -5.9599 | -1.5898 | -0.2 | -5.9599 | -1.5898 | -0.2 |
| H | -5.9791 | -3.7992 | -2.3523 | -5.9791 | -3.7992 | -2.3523 |
| H | -7.0846 | -2.4087 | -2.2943 | -7.0846 | -2.4087 | -2.2943 |
| H | -0.3668 | 10.7346 | -0.7633 | -0.3668 | 10.7346 | -0.7633 |
| H | -6.3804 | 2.6521 | 0.1841 | -6.3804 | 2.6521 | 0.1841 |
